# N-Centered Tripodal Phosphine Re(V) and Tc(V) Oxo Complexes: Revisiting a [3 + 2] Mixed-Ligand Approach

**DOI:** 10.1021/acs.inorgchem.2c00693

**Published:** 2022-05-11

**Authors:** Saul M. Cooper, Andrew J. P. White, Thomas R. Eykyn, Michelle T. Ma, Philip W. Miller, Nicholas J. Long

**Affiliations:** † Department of Chemistry, Imperial College London, Molecular Sciences Research Hub, 82 Wood Lane, White City Campus, London W12 0BZ, UK; ‡ School of Biomedical Engineering & Imaging Sciences, King’s College London, 4^th^ Floor Lambeth Wing, St Thomas’ Hospital, London SE1 7EH, UK

## Abstract

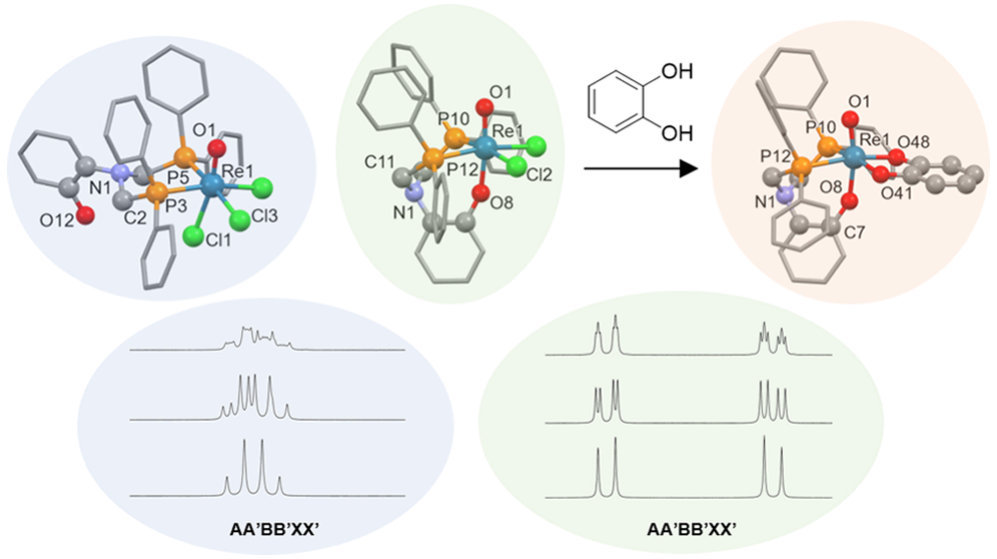

N-Triphos derivatives (NP_3_^R^, R = alkyl, aryl) and asymmetric variants (NP_2_^R^X^R′^, R′ = alkyl, aryl, X = OH, NR_2_, NRR′) are an underexplored class of tuneable, tripodal ligands in relation to the coordination chemistry of Re and Tc for biomedical applications. Mixed-ligand approaches are a flexible synthetic route to obtain Tc complexes of differing core structures and physicochemical properties. Reaction of the NP_3_^Ph^ ligand with the Re(V) oxo precursor [ReOCl_3_(PPh_3_)_2_] generated the bidentate complex [ReOCl_3_(κ^2^-NP_2_^Ph^OH^Ar^)], which possesses an unusual AA’BB’XX’ spin system with a characteristic second-order NMR lineshape that is sensitive to the bi- or tridentate nature of the coordinating diphosphine unit. The use of the asymmetric NP_2_^Ph^OH^Ar^ ligand resulted in the formation of both bidentate and tridentate products depending on the presence of base. The tridentate Re(V) complex [ReOCl_2_(κ^3^-NP_2_^Ph^O^Ar^)] has provided the basis of a new reactive “metal-fragment” for further functionalization in [3 + 2] mixed-ligand complexes. The synthesis of [3 + 2] complexes with catechol-based π-donors could also be achieved under one-pot, single-step conditions from Re(V) oxo precursors. Analogous complexes can also be synthesized from suitable ^99^Tc(V) precursors, and these complexes have been shown to exhibit highly similar structural properties through spectroscopic and chromatographic analysis. However, a tendency for the {M^V^O}^3+^ core to undergo hydrolysis to the {M^V^O_2_}^+^ core has been observed both in the case of M = Re and markedly for M = ^99^Tc complexes. It is likely that controlling this pathway will be critical to the generation of further stable Tc(V) derivatives.

## Abstract 2

An N-centered tripodal heterofunctionalized phosphine ligand was used to generate a reactive “metal-fragment” based on the {M^V^O}^3+^ (M = Re, ^99^Tc) core for the formation of mixed-ligand [3 + 2] complexes. Characteristic lineshapes arising from an AA’BB’XX’ spin system are diagnostic of bidentate vs tridentate coordination modes of the ligand.

## Introduction

The design of substitutionally inert rhenium and technetium metal-chelates for incorporation into targeted radiopharmaceuticals for molecular imaging and radiotherapy is an active area of research.^1−6^^99m^Tc is a γ-emitting radionuclide used routinely in single-photon emission computed tomography (SPECT), whereas its heavier congener Re has two β^*–*^-emitting radioisotopes, ^186^Re and ^188^Re, suitable for use in radiotherapy.^7,8^ Moreover, the potential use of these Re radioisotopes in conjunction with a ^99m^Tc imaging agent in a so-called “theranostic pair” is also of current interest, made possible by the physicochemical similarities arising from the isostructural nature of many Re and Tc complexes.^9,10^

Consequently, efforts have focused on developing new chelators that impart the necessary *in vivo* stability for these radionuclides to act as imaging or therapeutic agents, alongside possessing functionalities applicable to the synthesis of target-specific radiopharmaceuticals with known and reproducible biological activity.^11,12^ These prerequisites mean that bifunctional chelators (BFCs), which form the desired radiopharmaceutical under mild radiolabelling conditions with minimal isomerism and are compatible with a range of targeted biomolecules, are most desirable.^13^

**Chart 1:**
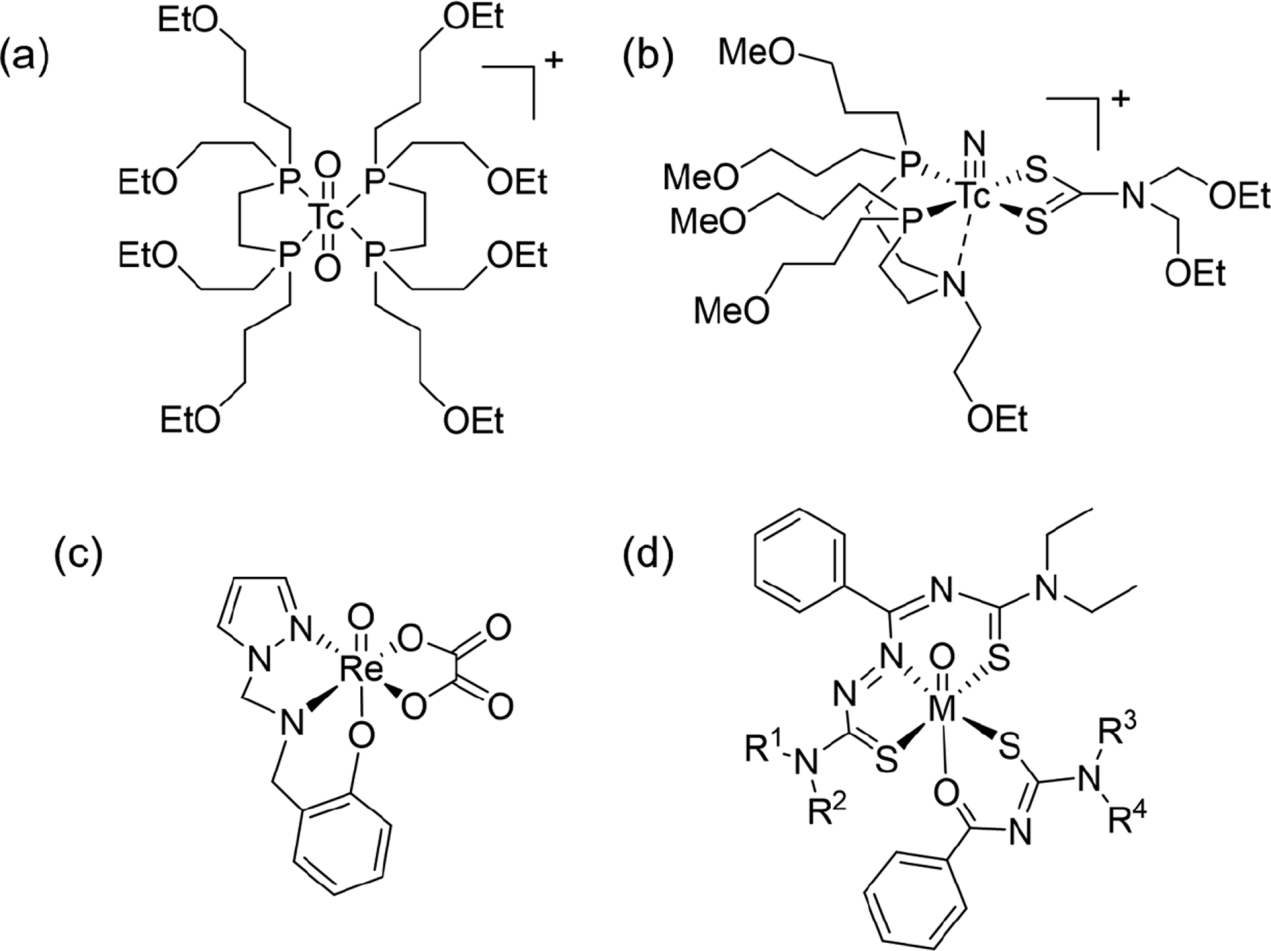
(a) [^99m^Tc]-[Tc-tetrofosmin]^+^,^28^ (b) [^99m^Tc]-[TcN(DBODC)(PNP5)]^+^,^69^ (c) [3 + 2] {M^V^O}^3+^ Complex Incorporating a Pyrazole-Containing Tridentate Ligand and Dianionic Bidentate Ligand,^63^ and (d) [3 + 2] {M^V^O}^3+^ Complexes with Thiosemicarbazones and Benzoylthioureas (M = Re, Tc)^67,68^

Phosphine ligands, including a range of heterofunctionalized variants with additional donor groups, are known to form kinetically inert metal complexes with both technetium and rhenium in a range of different oxidation states.^14−22^ Several monodentate and bidentate phosphine ligands have been used to coordinate ^99m^Tc effectively under radiochemical conditions appropriate for the formulation of water-soluble radiopharmaceuticals,^23−27^ the most notable example being the myocardial perfusion imaging agent [^99m^Tc]-[Tc-tetrofosmin] (Myoview), incorporating an ether-functionalized diphosphine (Chart 1a).^28^ However, further modification of these ligands to incorporate additional functional groups to facilitate target-specific imaging is frequently prohibited by the synthetic complexity necessary to adapt the carbon backbone and the sensitivity of many phosphines toward oxidation under aqueous conditions.^29,30^ A recent example published by Kama et al. employed a more facile synthesis to bis(diphenylphosphino)alkylamine ligands for bidentate coordination in water-soluble Re and ^99^Tc complexes.^31^

Tridentate phosphine ligands can provide additional stability to such transition-metal complexes, through exploitation of the chelate effect, but their usage has likewise been limited by the synthetic challenge they present, often requiring the use of highly sensitive metal phosphide reagents.^32^ In contrast, N-triphos (NP_3_^R^) ligands are a class of easily accessible nitrogen-centered triphosphine compounds that can be synthesized easily in a phosphorus-based Mannich reaction from the corresponding secondary phosphine.^33^ Asymmetric variants (NP_2_^R^X^R′^) are also readily accessible by replacement of ammonia in the above reaction with an appropriate primary amine.^34−36^ Previous work in our group has utilized the facial coordination of these ligands in the synthesis of hydrogenation catalysts for biomass-derived levulinic acid with ruthenium and other transition metals;^37−39^ investigation of the electronic and steric properties of several NP_3_^R^ ligands with tungsten;^40^ and dinitrogen coordination and activation using molybdenum and cobalt complexes.^41,42^

A highly successful strategy employing tridentate heterofunctionalized phosphine ligands as anchor ligands in the formation of mixed-ligand Re and Tc mono-nitrido complexes has been conducted by Tisato and co-workers.^43^ These PNP ligands, incorporating a five-membered nitrogen-bridged diethylene backbone, can be used to form stable M(V) complexes with Re and Tc nitrido groups, modulated by a weak but crucial interaction between the bridging nitrogen and the site *trans* to the nitrido group.^44^ Variation of the heteroatom in the bridging diphosphine ligand has been shown to have a significant effect on the relative stability, and interconversion, of meridional and facial isomers, of which only the latter is reactive toward bidentate nucleophiles.^45^ This reactive “metal-fragment” approach,^46^ in which the labile ligands *trans* to the phosphorus groups are readily replaced by functionalized bidentate π-donors (notably dithiocarbamates), has been translated successfully to ^99m^Tc radiolabelling conditions and used in the formulation of a novel myocardial imaging agent [^99m^Tc]-[TcN(DBODC)(PNP5)]^+^, with perfusion properties rivaling clinical agents (Chart 1b).^47,48^ Successful targeted approaches using bidentate peptidic co-ligands are also known, but the process remains inherently two-step, with formation of a weakly coordinated {Tc^V^N}^3+^ complex prior to introduction of the targeting ligand.^49,50^

In contrast, most clinical Tc(V) radiopharmaceuticals including a mono-oxo unit generally incorporate tetradentate N_*x*_S_(4–*x*)_ ligands in square pyramidal geometries.^11,51,52^ In the case of mixed-ligand [3 + 1] complexes, synthesized by Spies and co-workers, tridentate dithiol ligands and a monodentate thiol ligand have been used to synthesize a range of complexes with a large degree of variability in their structures.^53−55^ However, these technetium complexes have been shown to often have poor *in vivo* stability due to transchelation by biological thiols.^56,57^ By moving to alternative ligands to thiolates, a number of groups have explored a range of [3 + 2] ligand sets designed to confer additional stability to {Re^V^O}^3+^-containing complexes through omission of monodentate ligands (additional to an oxo unit). However, these sets have seen only limited success in, or indeed attempted translation to, ^99m^Tc radiopharmaceutical formulation. Notable examples with Re include the use of tridentate ligands derived from Schiff bases,^58^ diolates and dithiolates;^59−62^ N-heterocycles;^63,64^ and heteroscorpionates,^65^ but very few have used heterofunctionalized phosphines incorporated into tridentate ligands (Chart 1c).^16,66^ More recently, Abram and co-workers have published a series of {M^V^O}^3+^ complexes incorporating tridentate thiosemicarbazones and bidentate benzoylthioureas, which present a promising [3 + 2] strategy for use with technetium (Chart 1d).^67,68^

Our approach to produce a substitution-inert “metal-fragment” for the {M^V^O}^3+^ core is to revisit a [3 + 2] strategy with a new class of heterofunctionalized phosphine ligand for Re(V) and Tc(V). The NP_3_^R^ and NP_2_^R^X^R′^ ligands are employed as potentially tridentate chelators due to their perceived favorable properties for stabilizing {M^V^O}^3+^ complexes. These include the presence of strongly coordinating phosphine donors; a bridgehead structure predisposed toward facial coordination to a metal center to limit isomer formation, and, in the case of asymmetric variants, an oxygen donor to exploit the well-established preference for oxygen donors *trans* to the oxo group in such complexes.^70−72^ {M^V^O}^3+^ complexes of this nature are desirable due to the inherent ease of access of oxidometallates(V) from the pertechnetate anion [^99m^Tc]-[TcO_4_]^−^ with common reducing agents, such as SnCl_2_, and the potential extension of this approach to the “kit”-based formulation of ^99m^Tc radiopharmaceuticals.^73^ In a single previous study, NP_2_^R^X^R′^ ligands have been applied to [Re^I^(CO)_3_]^+^ units, albeit in a bidentate coordination mode, and without extension to radioactive analogues.^36^

**Chart 2:**
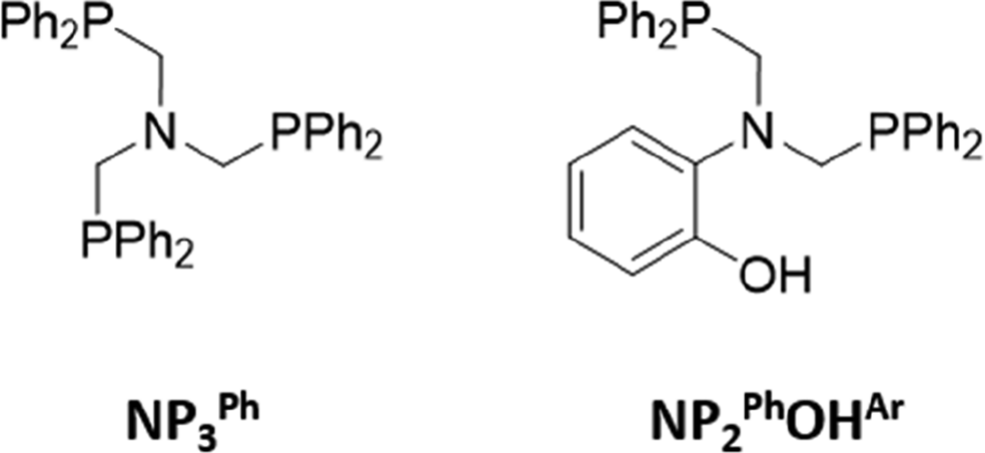
N-Centered Phosphine Ligands Used in This Study

Herein, the reactions between model phenyl-substituted phosphine ligands of this type (NP_3_^Ph^ and NP_2_^Ph^OH^Ar^ – Chart 2) and a number of {Re^V^O}^3+^ and {^99^Tc^V^O}^3+^ precursors are studied, and their products were characterized. In particular, the use of a phenolate functionality has been shown to facilitate facial tridentate coordination to {Re^V^O}^3+^ complexes^63^ and has been used here in the formation of the novel reactive “metal-fragment” complex, [ReOCl_2_(NP_2_^Ph^O^Ar^)] (**4**). The reactivity of this “metal-fragment” has also been explored with several bidentate π-donating ligands to produce stable [3 + 2] mixed-ligand Re(V) complexes. These [3 + 2] complexes have also been successfully synthesized through one-pot reactions from M(V) precursors, illustrating a preference for [3 + 2] heterocomplex formation, and extended to coordination chemistry with the long-lived ^99^Tc radioisotope.

## Experimental Section

### Materials

All reactions were performed under an N_2_ atmosphere using standard Schlenk techniques, unless otherwise stated. All further manipulations were performed in air. Dry solvents were obtained from an MBraun MB-SPS 800 Solvent Purification system, degassed by thoroughly sparging with nitrogen, and stored over activated 3 Å molecular sieves. NP_3_^Ph^,^33^ NP_2_^Ph^OH^Ar^,^34^ [ReOCl_3_(PPh_3_)_2_],^74^ (NBu_4_)[ReOCl_4_],^75^ and (NBu_4_)[TcOCl_4_]^76^ were prepared as described in the literature. Other reagents were commercially available and used as received. [^99^Tc]-(NH_4_)[TcO_4_] was kindly donated by Professor Philip Blower of Kings College London.

### Health Precautions

^99^Tc is a weak β^*–*^ emitter. All manipulations with this isotope were performed in a laboratory approved for the handling of radioactive materials. Normal glassware provides adequate shielding against low-energy β^*–*^ emission of the technetium compounds. Bremsstrahlung is not a significant problem due to the low energy of the β^*–*^ particles involved and the low amounts of ^99^Tc used. However, normal radiation safety procedures must be used at all times to prevent contamination and inhalation.

### Physical Measurements

^1^H, ^1^H{^31^P}, ^31^P{^1^H}, and ^13^C NMR spectra were acquired on Bruker AV-400, AV-500, or DRX-400 spectrometers. ^1^H, ^1^H{^31^P}, and ^31^P{^1^H} NMR spectra for samples containing ^99^Tc were acquired on a Bruker Avance III 400 spectrometer equipped with a BBO probe or a Bruker Avance III 700 spectrometer equipped with an AVIII console and a quadruple-resonance QCI cryoprobe. Chemical shifts are reported in ppm and referenced to the solvent for ^1^H and ^13^C{^1^H} NMR spectroscopy. ^31^P chemical shifts were referenced (δ = 0) externally to 85% H_3_PO_4_ (aq). Peak multiplicities are abbreviated as s = singlet, m = multiplet, d = doublet, t = triplet, q = quartet, qu = quintet, sx = sextet, spt = septet, dd = doublet of doublet, td = triplet of doublet, and br = broad. Mass spectrometry analyses were conducted by the Mass Spectrometry Service, Imperial College London. Infrared spectra were recorded on a PerkinElmer Spectrum FT-IR spectrometer. Flash silica column chromatography for Re compounds was performed on a Biotage Isolera Prime advanced automated flash purification unit using SNAP KP-Sil or Sfar Duo cartridges. Details of the single crystal X-ray diffraction (XRD) analysis can be found in the SI.

### Simulation of ^1^H NMR Lineshapes

NMR lineshapes were simulated in Matlab. Briefly, the Hamiltonian and density matrix for an AA’BB’XX’ system were calculated as the Kronecker tensor products of the corresponding Cartesian Pauli matrices *I_x_*, *I_y_*, *I_z_* and the identity matrix *E* to give a 64 × 64 matrix for a six-spin system taking care to retain the ordering of the spin indices 1–6. The Zeemann Hamiltonian for each spin was given by . Heteronuclear spins (^1^H and ^31^P) were assumed to be weakly coupled with a Hamiltonian given by . Homonuclear spins (^1^H and ^1^H′ or ^31^P and ^31^P′) were strongly coupled with a Hamiltonian given by  Evolution of the density matrix was calculated by numerical integration of the Liouville–von Neumann equation for an initial density operator  Thermal effects were neglected. The FID was calculated by taking the Trace of the density operator and the observable transverse magnetization Tr(σ(*t*)*I*^–^) followed by application of a phenomenological line broadening factor and Fourier transformation to yield the spectrum.

### Syntheses of Re Complexes

#### [*ReOCl_3_*(κ*^2^-NP_2_^Ph^P^Ph^*)] (Re-NP_3_) (**1**)

[ReOCl_3_(PPh_3_)_2_] (100 mg, 0.12 mmol) and NP_3_^Ph^ (74 mg, 0.12 mmol) were dissolved in toluene (30 mL) and heated to 100 °C for 18 h. Over this time, the reaction mixture turned from light green to dark green. The solvent was removed *in vacuo* to yield a dark green solid. This was redissolved in minimal CH_2_Cl_2_ and precipitated using hexane. The precipitate was collected by cannula filtration and the ^31^P{^1^H} NMR analysis indicated the presence of both **1** and its oxidized analogue **2**. Subsequent attempts to separate the complexes on silica column chromatography resulted in the oxidation of compound **1** to **2**. ^31^P{^1^H} (162 MHz, CDCl_3_): δ_P_ /ppm = – 29.4 (s, 2P, RPh_2_-*P*-Re), – 29.8 (s, 1P, RPh_2_*P*).

#### [*ReOCl_3_*(κ*^2^-NP_2_^Ph^P*(*O*)^*Ph*^)] (Re-NP_2_PO) (**2**)

Re-NP_2_PO was formed as a byproduct in the above reaction. Silica column chromatography (95:5 CH_2_Cl_2_:MeOH) allowed separation of the reaction products, and **2** was isolated as a pure dark blue solid (68 mg, 61%). Dark blue crystals suitable for X-ray analysis were grown by slow evaporation from CD_2_Cl_2_. ^1^H NMR (400 MHz, CD_2_Cl_2_): δ_H_ /ppm = 7.79–7.68 (m, 8H, C*H*^Ph^), 7.68–7.58 (m, 6H, C*H*^Ph^), 7.56–7.42 (m, 10H, C*H*^Ph^), 7.40–7.33 (m, 2H, C*H*^Ph^), 7.21–7.11 (m, 4H, C*H*^Ph^), 4.88 (m, 2H, N-C*H_2_*-P-Re), 4.20 (m, 2H, N-C*H_2_*-P-Re), 3.64 (d, 2H, 1 J_HP_ = 3.8 Hz, N-C*H_2_*-PO). ^13^C NMR (CD_2_Cl_2_): 135.2 (t), 133.7 (t), 133.0 (d), 132.9 (s, *C*^Ph^) 132.2 (s), 132.0 (s), 131.5 (s), 131.4 (s), 130.8 (s, *C*^Ph^) 129.7 (s), 129.6 (s), 128.8 (t), 59.9 (t), 59.4 (t). ^31^P{^1^H} (162 MHz, CD_2_Cl_2_): δ_P_ /ppm = + 25.3 (s, 1P, RPh_2_*P*=O), – 31.0 (s, 2H, RPh_2_*P*-Re). FT-IR (solid, cm^–1^): ν_Re=O_ = 989 (m), ν_PO_ = 1093 (s)*.* HR-ESMS: *m*/*z* calc. for [M-Cl] + at 900.0893, found at 900.0883.

#### [*ReOCl_2_*(κ*^2^-NP_2_^Ph^OH^Ar^*)] (Re-NP_2_OH) (**3**)

[ReOCl_3_(PPh_3_)_2_] (100 mg, 0.12 mmol) and NP_2_^Ph^OH (61 mg, 0.12 mmol) were dissolved in MeCN (30 mL) and heated to 60 °C for 18 h. Over this time, the reaction mixture turned from bright green to darker green. The solvent was removed *in vacuo*, and the green solid redissolved in minimal CH_2_Cl_2_ and precipitated from hexane (50 mL). The precipitate was collected by filtration and dried under high vacuum to give a pale green solid (87 mg, 87%). The product was found to be poorly soluble in chlorinated solvents but highly soluble in acetonitrile and methanol. Green crystals suitable for X-ray analysis were grown by vapor diffusion of acetone with hexane. ^1^H NMR (400 MHz, d^3^-MeCN): δ_H_ /ppm = 7.76–7.64 (m, 8H, C*H*^Ph^), 7.55–7.43 (m, 8H, C*H*^Ph^), 7.40–7.33 (m, 4H, C*H*^Ph^), 7.14 (ddd, 1H,^3^J_HH_ = 8.1 Hz,^3^J_HH_ = 7.4 Hz,^4^J_HH_ = 1.7 Hz, Ar-*H*), 7.10 (s, 1H, OH), 6.91 (dd, 1H,^3^J_HH_ = 8.1 Hz,^4^J_HH_ = 1.4 Hz, Ar-*H*), 6.73 (ddd, 1H,^3^J_HH_ = 7.8 Hz,^3^J_HH_ = 7.4 Hz,^4^J_HH_ = 1.4 Hz, Ar-*H*), 6.65 (dd, 1H,^3^J_HH_ = 7.8 Hz,^4^J_HH_ = 1.7 Hz, Ar-*H*), 4.99–4.86 (m, 4H, N-C*H_2_*-P-Re). ^31^P{^1^H} NMR (162 MHz, d^3^-MeCN): δ_P_ = – 29.1 (s, 2P, RPh_2_*P*-Re). FT-IR (solid, cm^–1^): ν_Re=O_ = 993 (s). HR-ESMS: *m/z* calc. for [M + Na]^+^ at 836.0194, found at 836.0201.

#### [*ReOCl_2_*(κ*^3^-NP_2_^Ph^O^Ar^*)] (Re-NP_2_O) (**4**)

Method A: [ReOCl_3_(PPh_3_)_2_] (200 mg, 0.24 mmol) and NP_2_^Ph^OH (121 mg, 0.24 mmol) were suspended in MeCN (30 mL). The reaction mixture was stirred for 10 min, and then DIPEA (0.037 mL, 0.26 mmol) added. The reaction mixture was then heated to 60 °C for 18 h, over which time the solution turned from bright green to brown. The reaction mixture was filtered, and the solvent was removed *in vacuo*. The resulting solid was dissolved in minimal CH_2_Cl_2_, precipitation was induced with hexane (50 mL), and the precipitate was collected by filtration. A pure yellow product was obtained by purification using silica column chromatography (95:5 CH_2_Cl_2_:MeOH) (125 mg, 67%). Crystals suitable for single-crystal X-ray diffraction were grown by vapor diffusion of CH_2_Cl_2_ with hexane.

Method B: **3** (50 mg, 0.062 mmol) was dissolved in dry and degassed MeCN (10 mL) stirred for 10 min at RT. DIPEA (0.065 mmol) was added, and the reaction mixture heated to 60 °C for 16 h. The solvent was removed *in vacuo*, and the crude residue purified by silica column chromatography (95:5 CH_2_Cl_2_:MeOH) to give the product as a yellow-brown solid (32 mg, 67%). ^1^H NMR (400 MHz, d^3^-MeCN): δ_H_ /ppm = 7.67–7.57 (m, 4H, C*H*^Ph^), 7.52–7.47 (m, 2H, C*H*^Ph^), 7.40–7.33 (m, 10H, C*H*^Ph^), 7.30–7.24 (m, 4H, C*H*^Ph^), 7.06 (ddd, 1H,^3^J_HH_ = 8.1 Hz,^3^J_HH_ = 7.4 Hz,^4^J_HH_ = 1.7 Hz, Ar-*H*), 7.00 (dd, 1H,^3^J_HH_ = 7.8 Hz,^4^J_HH_ = 1.7 Hz, Ar-*H*), 6.74 (ddd, 1H,^3^J_HH_ = 7.8 Hz,^3^J_HH_ = 7.4 Hz,^4^J_HH_ = 1.4 Hz, Ar-*H*), 6.59 (dd, 1H,^3^J_HH_ = 8.1 Hz,^4^J_HH_ = 1.4 Hz, Ar-*H*), 5.31–5.24 (m, 2H, N-C*H_2_*-P-Re), 5.03–4.96 (m, 2H, N-C*H_2_*-P-Re). ^13^C (101 MHz, CDCl_3_): δ_C_ /ppm = 138.8 (s), 138.3 (s), 134.0 (t), 133.0 (t), 131.7 (s), 131.0 (s), 129.7 (s), 129.0 (t), 128.9 (s) 128.4 (t), 122.9 (s), 122.6 (s), 58.9 (s). ^31^P{^1^H} NMR (162 MHz, d^3^-MeCN): δ_P_ /ppm = – 30.9 (s, 1P, RPh_2_P-Re). FT-IR (solid, cm^–1^): ν_Re=O_ = 972 (s). HR-ESMS: *m/z* calc. for [M + H]^+^ at 777.0608, found at 778.0617, *m/z* calc. for [M-Cl + MeCN]^+^ at 783.1107, found at 783.1094.

#### [*ReO_2_Cl*(κ*^2^-NP_2_^Ph^OH*)] (ReO_2_-Cl-NP_2_OH) (**5**)

Method A: **5** was obtained in a low yield as a byproduct from the preparation of **4**, described above. The pure product could be obtained following purification using silica column chromatography (90:10 CH_2_Cl_2_:MeOH) (42 mg, 23%).

Method B: **5** could also be obtained from hydrolysis of **3**. **3** (50 mg, 0.062 mmol) was dissolved in MeCN in air and stirred at RT for 10 min. DIPEA (0.065 mmol) was added, and the reaction mixture heated to 60 °C for 16 h. The solvent was removed *in vacuo*, and the crude mixture was purified by silica column chromatography (90:10 CH_2_Cl_2_:MeOH) to give the product as a brown solid (22 mg, 45%). ^1^H NMR (400 MHz, d^3^-MeOD): 7.45 (s, br, 8H, C*H*^Ph^), 7.32 (t,^3^J_HH_ = 7.5 Hz, 4H, C*H*^Ph^), 7.13 (t,^3^J_HH_ = 7.5 Hz, 8H, C*H*^Ph^), 6.87 (td,^3^J_HH_ = 7.6 Hz,^4^J_HH_ = 1.7 Hz, 1H, Ar-*H*), 6.64 (dd,^3^J_HH_ = 8.0 Hz,^4^J_HH_ = 1.4 Hz, 1H, Ar-*H*), 6.31 (td,^3^J_HH_ = 7.6 Hz,^4^J_HH_ = 1.4 Hz, 1H, Ar-*H*), 5.60 (dd,^3^J_HH_ = 8.0 Hz,^4^J_HH_ = 1.7 Hz, 1H, Ar-*H*), 4.36 (s, 4H, N-C*H_2_*-P-Re). ^31^P{^1^H} NMR (162 MHz, d^3^-MeOD): δ_P_ /ppm = – 36.7 (s, 1P, RPh_2_*P*-Re). FT-IR (solid, cm^–1^): ν_Re=O_ = 999 (m). HR-ESMS: *m/z* calc. for [M-Cl]^+^ at 724.1180, found at 724.1180.

### General Procedure for the Synthesis of [3 + 2] Complexes from **4**

**4** (20 mg, 0.025 mmol) was dissolved in CH_2_Cl_2_ (20 mL), and the bidentate ligand (catechol, oxalic acid, ethylene glycol, methyl 3,4-dihydroxyphenylacetate, 6,7-dihydroxycoumarin) (0.025 mmol) was added to the solution under N_2_. The reaction mixture was stirred for 10 min, after which two drops of triethylamine were added. The mixture was stirred at 60 °C for between 18 and 36 h and monitored by thin-layer chromatography (95:5 v:v CH_2_Cl_2_:MeOH). Upon disappearance of the starting material by TLC, the solvent was removed *in vacuo.* The brown residue was redissolved in minimal CH_2_Cl_2_ and precipitated using hexane (50 mL). The precipitate was collected and further purified by silica column chromatography (95:5 v:v CH_2_Cl_2_:MeOH) to give the products as dark brown/red solids.

#### [*ReO*(*cat-O,O*)(*NP_2_^Ph^O^Ar^*)] (Re-cat-O,O-NP_2_O) (**6**)

17 mg (82%). ^1^H NMR (400 MHz, CDCl_3_): δ_H_ /ppm = 7.83–7.74 (m, 4H, C*H*^Ph^), 7.47–7.26 (m, 16H, C*H*^Ph^), 7.04–6.98 (m, 2H, cat-*H*), 6.74 (1H, dd,^3^J_HH_ = 7.7 Hz,^4^J_HH_ = 1.7 Hz, Ar-*H*), 6.71–6.66 (m, 2H, cat-*H*), 6.63 (ddd, 1H,^3^J_HH_ = 8.1 Hz,^3^J_HH_ = 7.2 Hz, ^4^J_HH_ = 1.7 Hz, Ar-*H*), 6.34 (ddd, 1H,^3^J_HH_ = 7.7 Hz,^3^J_HH_ = 7.4 Hz, ^4^J_HH_ = 1.4 Hz, Ar-*H*), 6.29 (dd, 1H,^3^J_HH_ = 8.1 Hz, ^4^J_HH_ = 1.4 Hz Ar-*H*), 5.17–5.09 (m, 2H, N-C*H_2_*-P-Re), 4.88–4.79 (m, 2H, N-C*H_2_*-P-Re). ^13^C NMR (101 MHz, CDCl_3_): δ_C_ /ppm = 162.9 (s), 162.5 (s), 144.4 (s), 138.0 (s), 134.0 (t), 132.9 (t), 131.1 (s), 130.8 (s), 128.8 (t), 128.4 (t), 128.1 (s), 123.6 (s), 120.5 (s), 120.2 (s), 119.9 (s), 115.6 (s), 115.0 (s), 57.7 (t). ^31^P{^1^H} NMR (162 MHz, CDCl_3_): δ_P_ /ppm = – 32.9 (s, 1P, RPh_2_P-Re). HR-ESMS: *m/z* calc. for [M + H]^+^ at 816.1443, found at 816.1464.

#### [*ReO*(*Ox-O,O*)(*NP_2_^Ph^O^Ar^*)] (Re-Ox-O,O-NP_2_O) (**7**)

15 mg (74%). ^1^H NMR (400 MHz, CDCl_3_): δ_H_ /ppm = 7.61–7.43 (m, 6H, C*H*^Ph^), 7.43–7.28 (m, 12H, C*H*^Ph^), 6.89 (dd, 1H,^3^J_HH_ = 7.8 Hz,^4^J_HH_ = 1.7 Hz, Ar-*H*), 6.83 (ddd, 1H,^3^J_HH_ = 8.1 Hz,^3^J_HH_ = 7.4 Hz, ^4^J_HH_ = 1.7 Hz, Ar-*H*), 6.59 (ddd, 1H,^3^J_HH_ = 7.8 Hz,^3^J_HH_ = 7.4 Hz,^4^J_HH_ = 1.4 Hz, Ar*-H*), 6.47 (dd, 1H, ^3^J_HH_ = 8.1 Hz,^4^J_HH_ = 1.4 Hz, Ar-H), 5.23–5.14 (m, 2H, N-C*H_2_*-P-Re), 4.88–4.78 (m, 2H, N-C*H_2_*-P-Re). ^31^P{^1^H} NMR (162 MHz, CDCl_3_): δ_P_ /ppm = – 29.6 (s, 2P, RPh_2_*P*-Re). HR-ESMS: *m/z* calc. for [M + H]^+^ at 796.1028, found at 796.1050.

#### [*ReO*(*gly-O,O*)(*NP_2_^Ph^O^Ar^*)] (Re-gly-O,O-NP_2_O) (**8**)

12 mg (60%). ^1^H NMR (400 MHz, CD_2_Cl_2_): δ_H_ /ppm = 7.77–7.66 (m, 4H, C*H*^Ph^), 7.48–7.38 (m, 6H, C*H*^Ph^), 7.37–7.21 (m, 10H, C*H*^Ph^), 6.70 (dd, 1H,^3^J_HH_ = 7.7 Hz,^4^J_HH_ = 1.7 Hz, Ar*-H*), 6.66 (ddd, 1H,^3^J_HH_ = 8.1 Hz, ^3^J_HH_ = 7.3 Hz,^4^J_HH_ = 1.7 Hz, Ar*-H*), 6.31 (dd, 1H, ^3^J_HH_ = 8.1 Hz,^4^J_HH_ = 1.4 Hz, Ar*-H*), 6.24 (ddd, 1H,^3^J_HH_ = 7.7 Hz,^4^J_HH_ = 7.3 Hz,^4^J_HH_ = 1.4 Hz), 5.18–5.11 (m, 2H, N-C*H_2_*-P-Re), 4.92–4.84 (m, 2H, N-C*H_2_*-P-Re) 4.78–4.68 (m, 4H, O-C*H_2_*-C*H*_2_-O). ^31^P{^1^H} NMR (162 MHz, CD_2_Cl_2_): δ_P_ /ppm = – 37.1 (s, 2P, RPh_2_*P*-Re). HR-ESMS: *m/z* calc. for [M + H]^+^ at 768.1437, found at 768.1411.

#### [*ReO*(*cou-O,O*)(*NP_2_^Ph^O^Ar^*)] (Re-cou-O,O-NP_2_O) (**9**)

15 mg (64%): ^1^H NMR (400 MHz, CD_2_Cl_2_): δ_H_ /ppm = 7.76–7.66 (m, 4H, C*H*^Ph^), 7.63 (d, 1H,^3^J_HH_ = 9.4 Hz, C*H*^β^), 7.56–7.44 (m, 4H, C*H*^Ph^), 7.43–7.27 (m, 12H, C*H*^Ph^), 6.94 (s, 1H, C*H*^cat^), 6.92 (s, 1H, C*H*^cat^), 6.79 (dd,^3^J_HH_ = 7.7 Hz,^4^J_HH_ = 1.7 Hz, C*H*^ph-ol^), 6.68 (ddd, 1H,^3^J_HH_ = 8.1 Hz,^3^J_HH_ = 7.3 Hz,^4^J_HH_ = 1.7 Hz, C*H*^ph-ol^), 6.41 (ddd, 1H,^3^J_HH_ = 7.7 Hz,^3^J_HH_ = 7.3 Hz,^4^J_HH_ = 1.4 Hz, C*H*^ph-ol^), 6.22 (dd, 1H,^3^J_HH_ = 8.1 Hz,^4^J_HH_ = 1.4 Hz, C*H*^ph-ol^), 6.12 (d, 1H,^3^J_HH_ = 9.4 Hz, C*H*^α^), 5.28–5.20 (m, 1H, *CH_2_*), 5.09–4.91 (m, 1H, *CH_2_*), 4.80–4.71 (m, 1H, *CH_2_*). ^31^P{^1^H} NMR (162 MHz, CDCl_3_): δ_P_ /ppm = – 30.0 (d,^2^J_PP_ = 14 Hz, 1P, RPh_2_*P*-Re), – 33.3 (d,^2^J_PP_ = 14 Hz, 1P, RPh_2_*P*-Re). HR-ESMS: *m/z* calc. for [M + H]^+^ at 977.1719, found at 977.1723.

#### [*ReO*(*cat-O,O-COOMe*)(*NP_2_^Ph^O^Ar^*)] (Re-dhpma-O,O-NP_2_O) (**10**)

18 mg (79%). ^1^H NMR (400 MHz, CDCl_3_): δ_H_ /ppm = 7.82–7.73 (m, 4H, C*H*^Ph^), 7.46–7.37 (m, 8H, C*H*^Ph^), 7.36–7.24 (m, 12H, C*H*^Ph^), 6.95–6.90 (m, 2H, C*H*^Ph^), 6.76–6.72 (m, 1H, Ar-*H*), 6.67–6.58 (m, 2H, Ar-*H*), 6.38–6.30 (m, 2H, Ar-*H*), 5.17–5.08 (m, 2H, N-C*H_2_*-P-Re), 4.89–4.79 (m, 2H, N-C*H_2_*-P-Re), 3.78–3.73 (m, 2H, N-C*H_2_*-P-Re, 3.70 (s, 3H, R-COO-C*H_3_*), 3.65–3.60 (m, 2H, R-C*H_2_*-COOMe). ^31^P{^1^H} NMR (162 MHz, CDCl_3_): δ_P_ /ppm = – 32.7 (d,^2^J_PP_ = 14 Hz, 1P, RPh_2_*P*-Re), −32.9 (d,^2^J_PP_ = 14 Hz, 1P, RPh_2_*P*-Re). HR-ESMS: *m/z* calc. for [M + H]^+^ at 888.1654, found at 888.1636.

### General Procedure for the One-Pot Synthesis of [3 + 2] Complexes from [ReOCl_3_(PPh_3_)_2_]

[ReOCl_3_(PPh_3_)] (100 mg, 0.21 mmol), NP_2_^Ph^OH^Ar^ (0.21 mmol), and the bidentate ligand (2-mercaptophenol or *N*-acetyldopamine) (0.21 mmol) were dissolved in MeCN (30 mL) and stirred for 10 min. DIPEA (0.03 mL) was added by syringe under N_2_. The reaction mixture was heated to 60 °C for 16 h. Volatiles were removed *in vacuo*, the resultant residue was dissolved in minimal CH_2_Cl_2_, and the product mixture was precipitated using hexane. The brown precipitate was collected by filtration and purified by silica column chromatography (95:5 v:v CH_2_Cl_2_:MeOH) to give the pure product. Compounds **6**, **7**, and **10** were also prepared through this method.

#### [*ReO*(*cat-O,O-NHAc*)(κ*^3^-NP_2_^Ph^OH^Ar^*)] (Re-dop-O,O-NP_2_O) (**11**)

52 mg (34%) ^1^H NMR (400 MHz, CDCl_3_): δ_H_ /ppm = 7.87–7.74 (m, 4H, C*H*^Ph^), 7.53–7.30 (m, 16H, C*H*^Ph^), 6.95 (d,^3^J_HH_ = 7.8 Hz, 1H, cat-*H*), 6.86 (d,^4^J_HH_ = 2.0 Hz, 1H, cat-*H*), 6.78 (dd,^3^J_HH_ = 7.9 Hz,^4^J_HH_ = 1.7 Hz, 1H, Ar-*H*), 6.67 (ddd,^3^J_HH_ = 8.2 Hz,^3^J_HH_ = 7.4 Hz,^4^J_HH_ = 1.7 Hz, 1H, Ar-*H*), 6.52 (dd,^3^J_HH_ = 7.8 Hz,^4^J_HH_ = 2.0 Hz, 1H, cat-*H*), 6.38 (td,^3^J_HH_ = 7.4 Hz,^4^J_HH_ = 1.5 Hz, 1H, Ar-*H*), 6.33 (dd,^3^J_HH_ = 7.9 Hz,^4^J_HH_ = 1.5 Hz, 1H, cat-*H*), 5.55 (s, br, 1H, N*H*) 5.21–5.10 (m, 2H, N-C*H_2_*-P-Re), 4.95–4.81 (m, 2H, N-C*H*_2_-P-Re), 3.58 (q,^3^J_HH_ = 6.4 Hz, 2H, NH-C*H_2_*-CH_2_-Ar), 2.82 (q,^3^J_HH_ = 6.4 Hz, NH-CH_2_-C*H*_2_-Ar), 1.95 (s, 3H, C*H_3_*).). ^31^P{^1^H} NMR (162 MHz, CDCl_3_): δ_P_ /ppm = – 32.8 (s, br, 2P, RPh_2_*P*-Re). HR-ESMS: *m/z* calc. for [M + H]^+^ at 901.1970, found at 901.1982. *m/z* calc. for [M + Na]^+^ at 923.1789, found at 923.1816. *m/z* calc. for [M + K]^+^ at 939.1529, found at 939.1531.

#### [*ReO*(*ar-S,O*)(*NP_2_^Ph^O^Ar^*)] (Re-ar-S,O-NP_2_O) (**12**)

17 mg (12%). ^1^H NMR (CDCl_3_): δ_H_ /ppm = 7.85–7.77 (m, 2H, C*H*^Ph^), 7.70–7.62 (m, 2H, C*H*^Ph^), 7.54–7.46 (m, 4H, C*H*^Ph^), 7.44–7.22 (m, 14H, C*H*^Ph^), 6.93–6.86 (m, 2H, Ar*-H*), 6.79–6.76 (m, 1H, Ar*-H*), 6.75–6.69 (m, 2H, Ar*-H*), 6.43–6.35 (m, 2H, Ar*-H*), 5.24 (d,^2^J_HH_ = 15.7 Hz, 1H, N-C*H_2_*-P-Re), 5.04 (dd,^2^J_HH_ = 15.5 Hz,^3^J_HH_ = 5.0 Hz, N-C*H_2_*-P-Re), 4.84 (d,^2^J_HH_ = 15.5 Hz, 1H, N-C*H_2_*-P-Re), 4.76 (dd,^2^J_HH_ = 15.7 Hz,^3^J_HH_ = 5.0 Hz, N-C*H_2_*-P-Re). ^31^P{^1^H} NMR (CDCl_3_): δ_P_ /ppm = – 29.1 (d,^2^J_PP_ = 15.0 Hz, 1P, RPh_2_*P*-Re), – 40.6 (d,^2^J_PP_ = 15 Hz, 1P, RPh_2_*P*-Re). HR-ESMS: *m/z* calc. for [M + H]^+^ at 832.1214, found at 832.1195.

#### [*TcOCl_3_*(κ*^2^-NP_2_^Ph^OH^Ar^*)] (Tc-NP_2_OH) (**Tc-3**)

(NBu_4_)[TcOCl_4_] (10 mg, 0.02 mmol) and NP_2_^Ph^OH^Ar^ (1 equiv) were added to a sealed glass vial equipped with a stirrer bar and purged under N_2_. MeCN (1 mL) was added under N_2_, and the vial was heated to 60 °C for 2 h with a vent needle. The vial was opened, and volatiles were removed under a jet of N_2_. The residue was dissolved in minimal CH_2_Cl_2_, precipitated using hexane (25 mL), and collected by centrifugation. This process was repeated three times to yield the product mixture as a purple solid. The entire sample was dissolved in d^3^-MeCN (600 μL) analyzed by NMR spectroscopy. **Tc-3** was confirmed as the major species in the reaction mixture. Selected analytical data; ^1^H NMR (400 MHz, d^3^-MeCN): δ_H_ /ppm = 5.13–5.04 (m, 2H, N-C*H_2_*-P-Re), 4.87–4.74 (m, 2H, N-C*H_2_*-P-Tc). ^31^P{^1^H} NMR (CDCl_3_): δ_P_ /ppm = + 27.1 (s, 2P, RPh_2_*P*-Tc).

#### [*TcO*(*cat-O,O*)(κ*^3^-NP_2_^Ph^O^Ar^*)] (Tc-cat-O,O-NP_2_O) (**Tc-6**)

(NBu_4_)[TcOCl_4_] (10 mg, 0.02 mmol), NP_2_^Ph^OH^Ar^ (1 equiv), and catechol (1.1 equiv) were added to a sealed glass vial equipped with a stirrer bar and purged under N_2_. MeCN (1 mL) was added under N_2_ followed by NEt_3_ (3 equiv), and the vial heated to 60 °C for 2 h with a vent needle. The vial was opened, and volatiles were removed under a jet of N_2_. The residue was dissolved in minimal CH_2_Cl_2_, precipitated using hexane (25 mL), and collected by centrifugation. This process was repeated three times to yield the product mixture as a purple solid. The presence of **Tc-6** could be postulated by TLC (2% MeOH in CH_2_Cl_2_, *R*_f_ = 1.0, see ESI), ^1^H NMR spectroscopy and low-resolution mass spectrometry data (see text).

#### [*TcO_2_Cl*(κ*^2^-NP_2_^Ph^OH^Ar^*)] (TcO_2_-NP_2_OH) (**Tc-5**)

The presence of **Tc-5** in both of the above reactions could be inferred by ^1^H NMR spectroscopy. Selected analytical data; ^1^H NMR (400 MHz, d^3^-MeCN): δ_H_ /ppm = 4.31 (d,^2^J_HP_ = 2.1 Hz, 4H, N-C*H_2_*-P-Re). ^31^P{^1^H} NMR (CDCl_3_): δ_P_ /ppm = + 26.9 (s, 2P, RPh_2_*P*-Tc).

## Results and Discussion

### Synthesis and Characterization of NP_3_^Ph^ Complexes

The phenyl-substituted N-triphos ligand, NP_3_^Ph^, was prepared according to the published method.^37^ This ligand was selected due to its ease of synthesis and its known propensity toward forming facial κ^3^-coordination geometries with transition metals in a number of different oxidation states.^38,40,41^

**Scheme 1:**
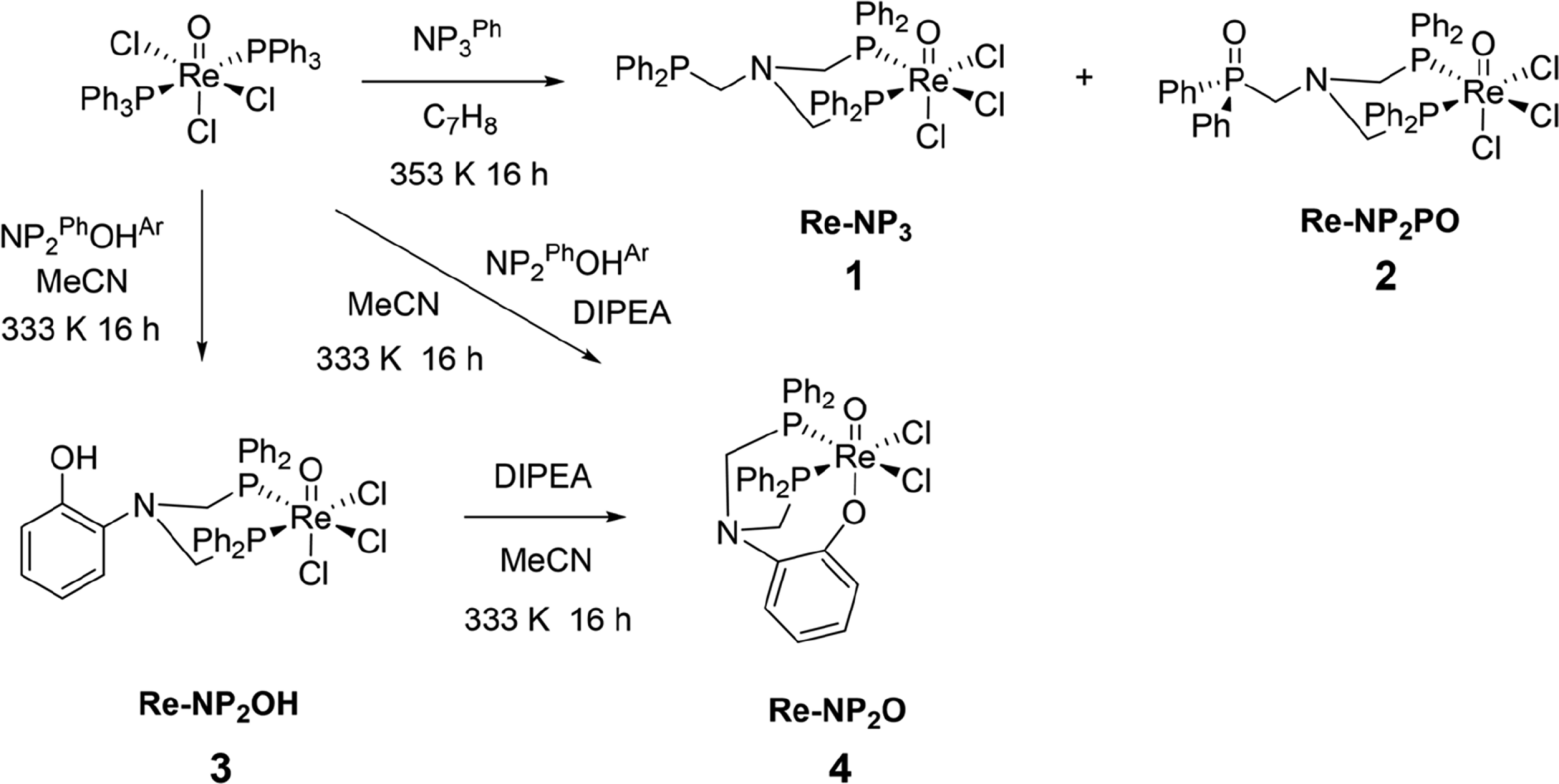
Synthesis of {Re^V^O}^3+^ Complexes Bearing NP_3_^Ph^ and NP_2_^Ph^OH^Ar^ Ligands

Scheme 1 illustrates the reactions performed between the commonly used Re(V) precursor [ReOCl_3_(PPh_3_)_2_] and the NP_2_^Ph^X^R^ ligands. Reaction of NP_3_^Ph^ with [ReOCl_3_(PPh_3_)_2_] in toluene at 373 K resulted in the formation of the diamagnetic complex [ReOCl_3_(κ^2^-NP_2_^Ph^P^Ph^)] (**1**), in which two PPh_3_ ligands have been displaced by one unit of NP_3_^Ph^. During workup of **1**, a second Re species was also produced, [ReOCl_3_(κ^2^-NP_2_^Ph^P(*O*)^Ph^)] (**2**), resulting from the oxidation of the free third phosphine arm of the coordinated NP_3_^Ph^ ligand. The oxidized arm displayed a singlet at δ_P_ = + 25.3 ppm (relative to the uncoordinated phosphine arm in **1** at δ_P_ = – 29.8 ppm) in the ^31^P{^1^H} NMR spectrum; a P=O absorption was observed at 1093 cm^–1^ in the FT-IR spectrum.

**Figure 1:**
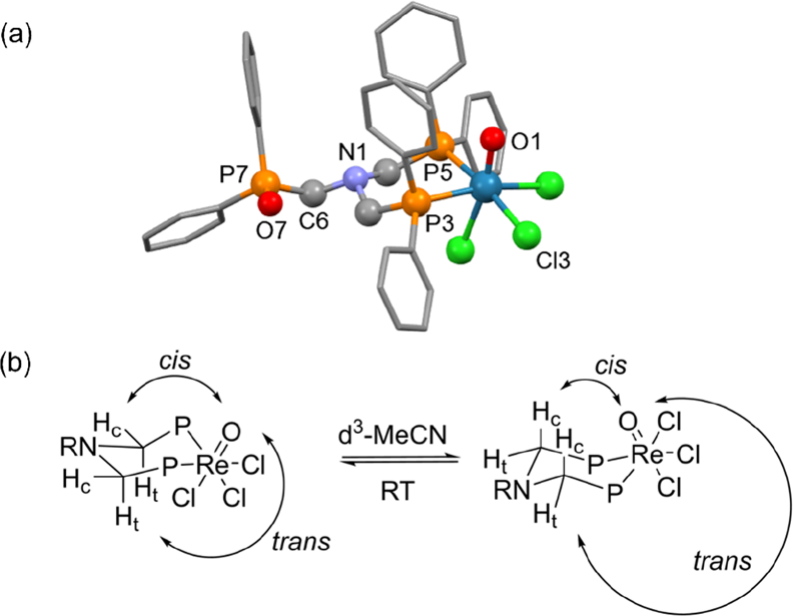
(a) Crystal structure of [ReOCl_3_(κ^2^-NP_2_^Ph^P(*O*)^Ph^)] (**2**). Hydrogens and a molecule of acetone have been omitted for clarity. (b) Ring conversion in Re–NP_2_X complexes illustrating the chemically inequivalent *cis* (H_c_) and *trans* (H_t_) hydrogens of the methylene group, differentiated by their orientation in relation to the [Re=O] bond. Interconversion (conformational exchange) between the two conformers is anticipated to occur readily in solution for the bidentate ligands but will become conformationally locked for the tridentate ligands.

Attempts to separate these two species by silica column chromatography resulted in further conversion of **1** to **2**. Nevertheless, crystallographic characterization enabled confirmation of the structure of **2**, in which the NP_3_^Ph^ ligand coordinates in a bidentate fashion to the metal center, alongside three chloride ligands to complete the pseudo-octahedral coordination sphere. Single crystals of **2** were obtained by slow evaporation of a CDCl_3_ solution of the complex (Figure 1a). The *cis* geometry of the phosphine donors is confirmed, as anticipated by the steric constraints imposed by the length of the bridge between the two phosphorus groups. The crystal structure exhibits an elongated Re–Cl (2.4403(17) Å) bond *trans* to the oxo group, relative to the equatorial Re–Cl bond (2.3737(17) Å), which is typical of rhenium oxo complexes due to the substantial *trans* influence of the oxo donor.^77^

The diamagnetic profile of these complexes is consistent with a low-spin d^2^ distorted octahedral geometry, which is commonly observed with Group 7 M(V) complexes containing an oxo unit.^78^ Therefore, ^1^H and ^31^P{^1^H} NMR spectroscopy can be used to effectively characterize these species in solution. The ^1^H NMR spectrum exhibits distinctive multiplets in the δ_H_ = 3.5–4.5 ppm region of the spectrum corresponding to the chemically inequivalent methylene protons of the coordinated ligand. Upon coordination of the phosphines to the Re metal center, a six-membered chelate is formed, as confirmed by the chair conformation of this ring observed in the crystal structure of **2** (Figure 1a). The geminal hydrogens of the bridging methylene units are rendered chemically inequivalent by their *cis* and *trans* orientations relative to the [Re=O] bond. The ring is expected to be conformationally flexible at room temperature due to the presence of a nitrogen atom allowing rapid inversion of the ring in solution, resulting in an average ^1^H NMR signal for the two conformers (Figure 1b). Nevertheless, the distinction between these fluxional geminal *cis* and *trans* hydrogens is retained upon this interconversion.

The ^1^H lineshapes for these multiplets arise from the combination of a ^2^J_HH_ geminal coupling between the methylene protons, two ^2^J_PH_ couplings to the neighboring phosphorus atom, and a further passive ^2^J_PP_ coupling between the coordinated phosphines, which introduces considerable second-order effects. Such behavior corresponds to an AA’BB’XX’ spin system for the N-(CH_2_-PPh_2_)_2_-Re- unit. The unusual nature of the lineshapes observed in all the{M^V^O}^3+^ complexes synthesized in this study led us to explore the spin system in greater detail through NMR simulations, which will be discussed *vide infra*.

^31^P{^1^H} NMR spectra for the complexes show characteristic singlets, reflecting the equivalent phosphorus environments that arise from the *C_s_* symmetry of the molecules. The coordinated phosphine groups of **1** exhibit a singlet at δ_P_ = −29.8 ppm and the free phosphine arm at δ_P_ = −29.4 ppm in a 2:1 integral ratio, indicating two different phosphorus environments within the molecule. **2** exhibits a singlet at δ_P_ = −30.6 ppm for the coordinated phosphine groups, a very slight lower frequency shift relative to its unoxidized analogue, and a singlet at δ_P_ = +25.3 ppm for the phosphine oxide unit.

Tridentate coordination of NP_3_^Ph^ in the above complexes is likely precluded by both the strong *trans* influence of the oxo donor enhancing the lability of the site opposite to it and the documented preference of this site for hard-type donors.^78^ Attempts to promote tridentate coordination of the NP_3_^Ph^ ligand, including the use of more polar solvents, elevated temperatures, and increased reaction times, were unsuccessful and either returned **1** or decomposition products.

### Synthesis and Characterization of a Bidentate Re(V) NP_2_^Ph^OH^Ar^ Complex

In order to facilitate tridentate coordination to the metal center, the asymmetric ligand NP_2_^Ph^OH^Ar^ was synthesized according to the literature.^34^ Smith and co-workers have successfully coordinated this ligand in a bidentate mode to a number of transition metals,^79,80^ but tridentate coordination of this ligand has not been observed prior to this study. This ligand, containing a phenol unit as a coordinating third arm, was envisioned to promote tridentate coordination to rhenium through deprotonation and coordination of the anionic oxygen donor to the coordination site *trans* to the oxo group. The extraordinary preference of this site for anionic oxygen donors is well-documented, and stability of complexes containing oxygen at this site is likely promoted through the presence of a stabilizing interaction of π symmetry across the [O=Re-OR]^2+^ fragment.^71,72^

**Figure 2:**
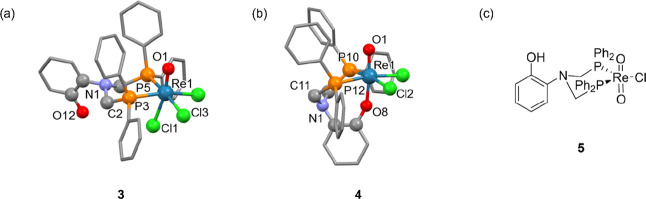
(a) Crystal structure for [ReOCl_3_(κ^2^-NP_2_^Ph^OH^Ar^)] (**3**). (b) Crystal structure for [ReOCl_2_(κ^3^-NP_2_^Ph^O^Ar^)] (**4**). Hydrogens have been omitted for clarity. (c) Line drawing of [ReO_2_Cl(κ^2^-NP_2_^Ph^OH^Ar^)] (**5**).

Reaction between NP_2_^Ph^OH^Ar^ and [ReOCl_3_(PPh_3_)_2_] in toluene, in the absence of base, resulted in formation of the diamagnetic d^2^ complex [ReOCl_3_(κ^2^-NP_2_^Ph^OH^Ar^)] (**3**), which could be purified and isolated by silica column chromatography. This complex had low solubility in chlorinated solvents but reasonable solubility in MeOH and MeCN, most likely due to the presence of the polar free phenol arm. Crystallographic characterization of the species confirmed the *cis* bidentate coordination of the phosphine groups and the protonated phenolic arm (Figure 2a).

The reaction between NP_2_^Ph^OH^Ar^ and the more labile Re(V) precursor (NBu_4_)[ReOCl_4_] in d^3^-MeCN was also conducted and was expected to proceed without the need for elevated temperatures. Indeed, this reaction, conducted at room temperature, also produced **3** in very good yield and high purity, as determined by ^1^H and ^31^P{^1^H} NMR spectroscopy. This was considered advantageous for the translation of such chemistry to ^99^Tc, as the complex [TcOCl_3_(PPh_3_)_2_] is unknown, whereas (NBu_4_)[TcOCl_4_] can be synthesized readily from [TcO_4_]^−^ through the action of concentrated hydrochloric acid.^76^

### Synthesis and Characterization of a Tridentate Re(V) NP_2_^Ph^O^Ar^ Complex

The reaction between NP_2_^Ph^OH^Ar^ and [ReOCl_3_(PPh_3_)_2_] in the presence of a tertiary amine base (such as NEt_3_ or DIPEA) conducted in MeCN at 60 °C resulted in the formation of the diamagnetic air-stable complex [ReOCl_2_(NP_2_^Ph^O^Ar^)] (**4**). This complex could be purified by silica column chromatography in CH_2_Cl_2_:MeOH and produces a deep yellow color when dissolved in organic solvents. X-ray analysis of single crystals of **4** grown by vapor diffusion of CH_2_Cl_2_ with hexane confirmed the tridentate coordination mode of the [P,P,O] donor ligand. X-ray crystal analysis illustrated the reciprocal *trans* configuration of the oxygen atoms, and the *boat*-like conformation adopted by the six-membered ring upon coordination of the phosphorus atoms to the metal center (Figure 2b). The O–Re–O angle for the [O=Re–O–Ar]^2+^ unit has a value of 173.19(19)°, close to 180°, reflecting the considerable π interaction across the dioxygen unit.^81^ Perhaps more telling of the extent of the π interactions in this unit is the C–O–Re angle, which reflects the distortion away from the idealized angle for sp^*2*^ hybridization (120°). This angle has a value of 156.8(4)°, a considerable distortion toward linearity. This distortion toward sp hybridization likely enables the 2p orbitals of the oxygen atom to participate in bonding orbitals encompassing the whole [O=Re–O–Ar]^2+^ unit. This distortion is likewise present in the synthesized [3 + 2] complexes. Table 1 contains selected bond lengths and bond angles for a range of {Re^V^O}^3+^ complexes synthesized in this study.

**Table 1:** Selected Bond Lengths (Å) and Bond Angles (°) for Re(V) Complexes

	Re–NP_2_PO (**2**)	Re–NP_2_OH (**3**)	Re–NP_2_O (**4**)	Re–cat-O,O-NP_2_O (**6**)	Re–ox-O,O-NP_2_O (**7**)
Re1–O1	1.679(5)	1.669(3)	1.684(4)	1.693(3)	1.683(3)
Re1–P3/P10	2.4389(18)	2.4626(12)	2.4422(14)	2.4378(13)	2.4299(10)
Re1–P5/P12	2.4580(17)	2.4445(12)	2.4621(15)	2.4815(15	2.4596(10)
Re1–Cl1	2.4403(17)	2.4246(11)			
Re1–Cl2	2.3739(18)	2.3655(12)	2.3920(17)		
Re1–Cl3	2.3737(17)	2.4142(12)	2.4165(12)		
Re1–O8			1.932(4)	1.974(3)	1.941(3)
Re1–O41/40				2.009(3)	2.057(3)
Re1–O48/43				2.037(4)	2.072(3)
P3–Re1–P5	86.04(6)	90.00(4)	89.16(5)	87.81(5)	87.64(3)
O1–Re1–Cl1	168.55(16)	168.50(11)			
O1–Re1–O8			173.19(19)	168.54(16)	171.82(13)
C7–O8–Re1			156.8(4)	146.2(3)	151.2(3)

**Figure 3:**
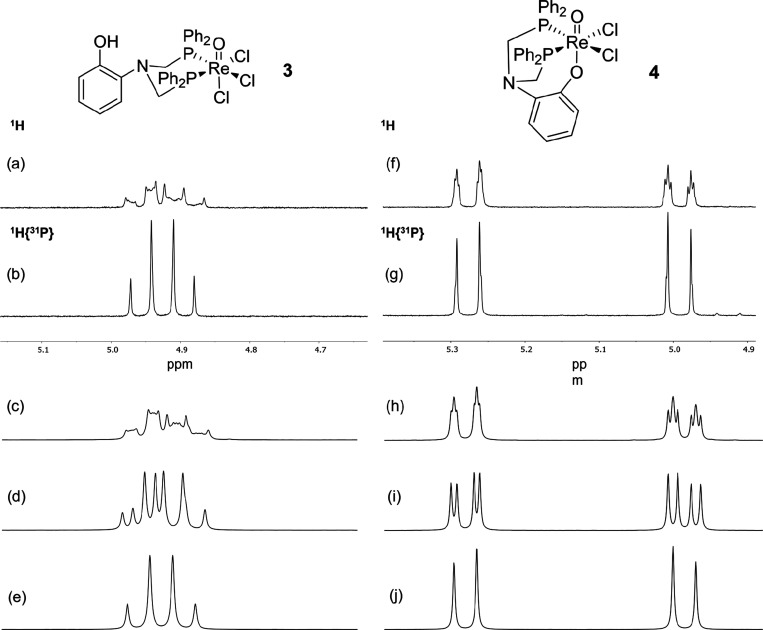
^1^H and ^1^H{^31^P} NMR spectra in d^3^-MeCN of the methylene region of complex **3** (a,b) (5.15–4.65 ppm region) and **4** (f,g) (5.35–4.90 ppm region). Full NMR spectra can be found in the SI. (c) Simulated lineshape of the ^1^H spectrum in (a). Parameters used in the simulation were ^2^J_HH’_ = 15.9 Hz; ^2^J_HP_ = 7 Hz; ^2^J_H′P_ = 14 Hz; ^2^J_PP′_ = 20 Hz; ω_1_ = 200 Hz; ω_2_ = 228 Hz; lb = 8 Hz. (d) Same parameters as in panel (c) but with ^2^J_PP′_ = 0 Hz. (e) Same parameters as in panel (c) but with ^31^P decoupling ^2^J_HP_ = ^2^J_H′P_ = 0 Hz. (h) Simulated lineshape of the ^1^H spectrum in panel (f). Parameters used in the simulation were ^2^J_HH′_ = 14 Hz; ^2^J_HP_ = 3 Hz; ^2^J_H′P_ = 5 Hz; ^2^J_PP′_ = 20 Hz; ω_1_ = 200 Hz; ω_2_ = 314 Hz; lb = 4 Hz. (i) Same parameters as in panel (h) but with ^2^J_PP′_ = 0 Hz. (j) Same parameters as in panel (h) but with ^31^P decoupling ^2^J_HP_ = ^2^J_H′P_ = 0 Hz.

The change in configuration of the six-membered chelate resulted in a marked change in the lineshapes of the diagnostic methylene region of the ^1^H NMR spectrum. Whereas in the chair-like conformation of the bidentate complexes, the resonances corresponding to the methylene carbons of the complex exhibit a highly distorted line shape with significant second-order couplings, the tridentate **4** complex instead exhibits smaller ^2^J_HP_ coupling constants. The variation in magnitude of this coupling constant inducing changes in the lineshapes has been further explored through NMR simulations (Figure 3). The lineshapes corresponding to the methylene protons in **4** consequently bear a stronger resemblance to a pair of doublets, in which the geminal ^2^J_HH_ coupling is the dominant interaction.

The lineshapes in this region of the ^1^H spectrum, hereafter referred to as the methylene region, are particularly diagnostic for interpreting the coordination mode of the ligand. As can be seen in Figure 3, for the case of **3**, ^31^P decoupling gives a simplified spectrum in which the geminal coupling between the *cis* and *trans* protons is clearly resolved (with roofing due to the proximity in chemical shift of the two environments). The measured value of ^2^J_HH_ in the case of **3** was found to be ^2^J_HH_ = 15.0 Hz. In the case of **4**, a smaller coupling is removed, one which has induced a smaller splitting compared to the non-decoupled case. The geminal coupling in **4** was measured as ^2^J_HH_ = 15.4 Hz. The difference in lineshape between Figure 3a (measured) and Figure 3e (simulated with ^2^J_PP’_ = 0) clearly suggests the presence of a strong, passive coupling between the magnetically inequivalent *cis*-configured phosphorus atoms.

**4** could also be synthesized by initial deprotonation of NP_2_^Ph^OH^Ar^ in MeCN solution using 1 equiv of KO^t^Bu. The progress of this deprotonation could be monitored visually by a color change from colorless to green. Addition of the solution containing the deprotonated ligand to a solution of [ReOCl_3_(PPh_3_)_2_] and subsequent heating of the reaction mixture resulted in formation of **4** but in a reduced yield due to the significant formation of a byproduct, the identity of which is likely to be the dioxo Re(V) complex [ReO_2_Cl(NP_2_^Ph^OH^Ar^)] (**5**) (Figure 2c). Notable features of this complex include a lower frequency phosphorus singlet chemical shift at δ_P_ = −36.7 ppm, at ∼10 ppm lower relative to the {Re^V^O}^3+^ complexes isolated. Additionally, the methylene region of the ^1^H NMR spectrum no longer contains multiple chemically inequivalent *cis* and *trans* hydrogens of the AA’BB’XX’ spin system but rather a lone singlet at δ_H_ = +4.39 ppm for chemically equivalent methylene hydrogens due to the {Re=O} *trans* to both environments. The dioxo complex byproduct [ReO_2_Cl(κ^2^-NP_2_^Ph^OH^Ar^)] (**5**) was identified by the presence of a molecular ion peak for [M-Cl]^+^ in the ES mass spectrum at *m/z* = 724.1180 (expected at *m/z* = 724.1180). While this does not confirm the presence of a chloride in the coordination sphere, it does strongly suggest a dioxo unit with a single NP_2_^Ph^OH^Ar^ ligand coordinated.

The reaction between NP_2_^Ph^OH^Ar^ and (NBu_4_)[ReOCl_4_] in the presence of base was also conducted in the hope of observing formation of **4**. However, this reaction generally resulted in a crude mixture of products in which varying proportions of **3**, **4**, and **5** could be observed by ^31^P{^1^H} NMR analysis. A cleaner one-pot variant of this reaction, achieved through the addition of a bidentate dioxygen ligand to the reaction mixture, was able to circumvent this issue of multiple product formation and is discussed in the next section.

When **3** was heated in MeCN at 60 °C overnight in the presence of NEt_3_, then the identity of the product formed was dependent on the presence of water in the system. Use of anhydrous solvents resulted in the formation of **4** as the major species; however, the use of standard grade solvents results in the formation of **5**, likely due to hydrolysis of the [O=Re-Cl]^2+^ unit.^82^ This effect has been observed as even greater in the case of ^99^Tc complexes and is discussed *vide infra*.

### Reactivity of [ReOCl_2_(NP_2_^Ph^O^Ar^)] with Bidentate Ligands

The {M^V^N(PNP)}^2+^ (M = Re, ^99m^Tc) “metal-fragment” is often combined with a “soft” sulfur-based donor in the bidentate component of mixed-ligand [3 + 2] complexes.^43^ Conversely, for {M^V^O}^3+^ cores, “hard” oxygen donors such as catechols, 1,2-diols, and 1,2-dicarboxylic acids are often preferred.^78^ In the case of the tridentate scorpionates dithiolates have been used to form [3 + 2] complexes with the [Re^V^O]^3+^ core.^83,84^ These ligands have been shown to be compatible with β-diketones and diamines, although with only bidentate coordination of the scorpionate exhibited in these latter cases.^85,86^ Papadopoulos and co-workers have used imino-pyridines as bidentate ligands to form complexes with an [S,N,O]/[N,N] donor set.^62^ Pyrazole-derived [N,N,O] ligands have been shown to stabilize [3 + 2] complexes with catechol and ethylene glycol,^63^ while [3 + 2] complexes of the closely related pyridyl-derived [N,N,O] ligand published by Abrahams et al. have been stabilized using an oxalic acid ligand.^87^ The trio of catechol, ethylene glycol, and oxalic acid has been used effectively with a number of different tridentate ligands and were selected as the starting point for exploring further ligand substitution chemistry with **4**.^64,65,88^ More specifically, a study using catechol as a bidentate ligand alongside a heterofunctionalized phosphine was conducted by Sigouin et al.^89^ The coordination behavior of [Re^V^O]^3+^ with dppe and catechol was explored, with the catechol only observed as binding to the metal center in the equatorial plane; the site *trans* to the oxo group was instead occupied by a halide ion. This suggests that the catechol is not expected to compete for binding to the labile *trans* site, despite phenolic oxygens being known to have an affinity for this position. A strongly π-donating dioxygen ligand may be able to better accommodate π-acceptor phosphine ligands with which it will be sharing d orbitals in the equatorial plane.

**Figure 4:**
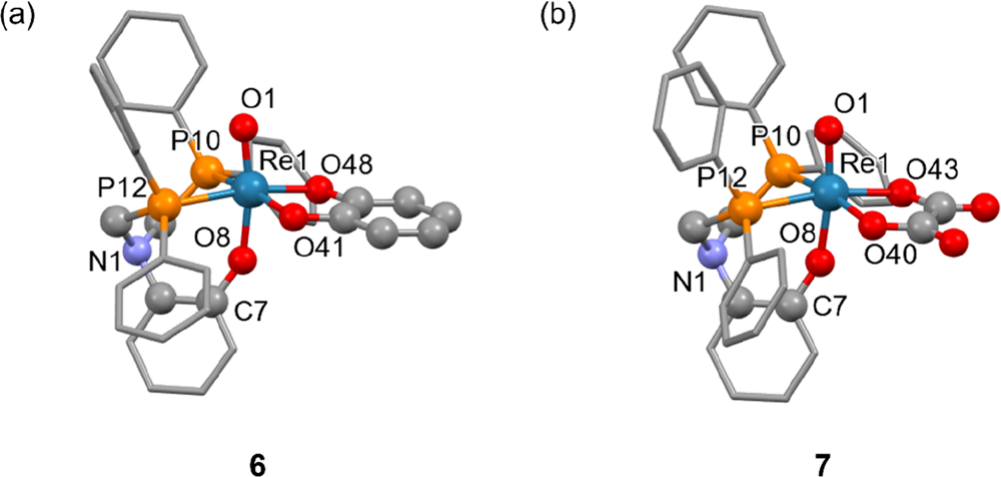
(a) Crystal structure of [ReO(cat-O,O)(κ^3^-NP_2_^Ph^O^Ar^)] (**6**) and (b) [ReO(ox-O,O)(κ^3^-NP_2_^Ph^O^Ar^)] (**7**). Hydrogens and co-crystallized solvent molecules have been omitted for clarity.

**Scheme 2:**
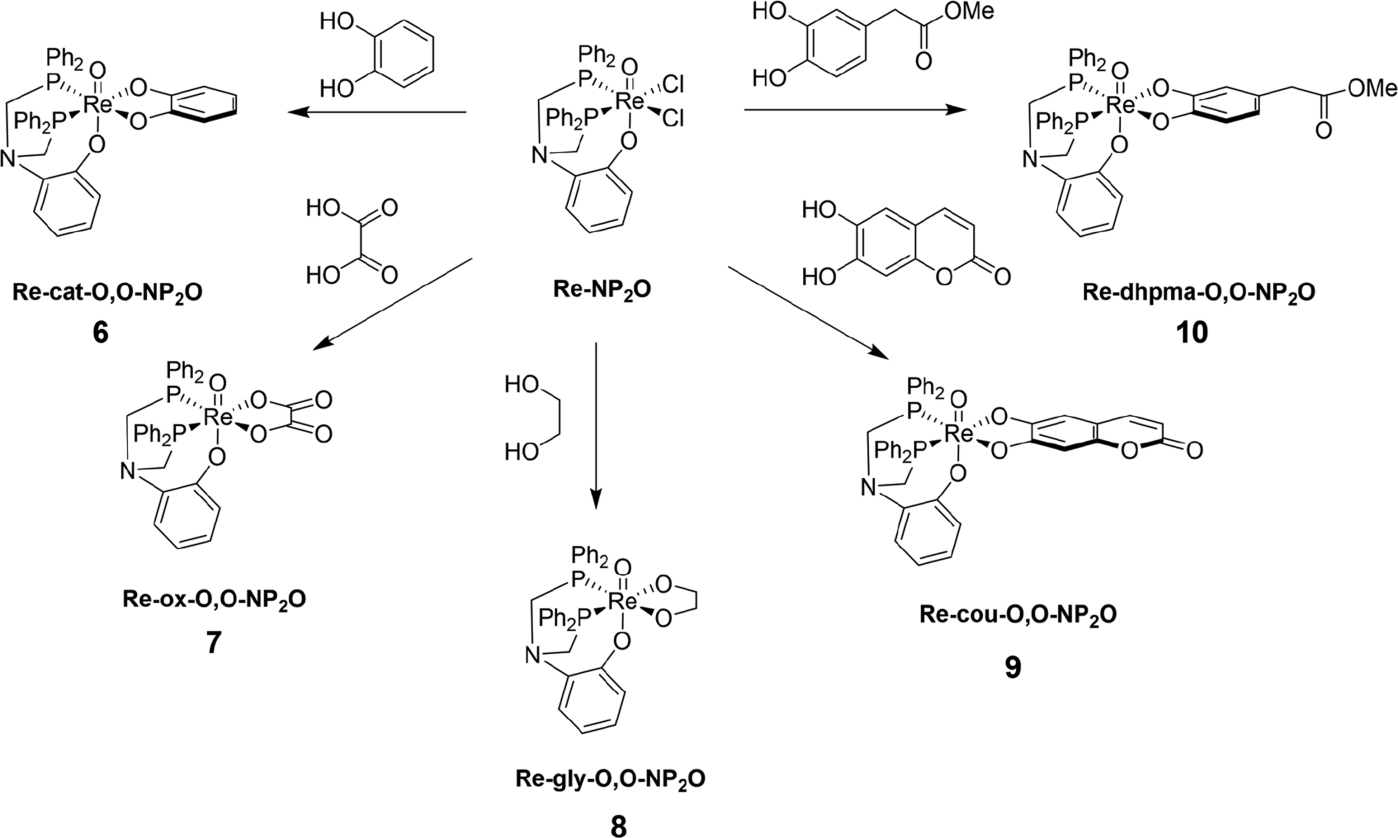
Synthesis of [3 + 2] Complexes from **4** Using Bidentate Oxygen-Donor Ligands

Scheme 2 depicts the range of [3 + 2] complexes synthesized via **4**. When **4** was dissolved in MeCN and heated in the presence of one equivalent of catechol and three equivalents of DIPEA, the complex [ReO(cat-O,O)(NP_2_^Ph^O^Ar^)] (**6**) was formed as the major product and could be purified by silica column chromatography in good yield. This air-stable diamagnetic [3 + 2] mixed-ligand complex displayed a single resonance in its ^31^P{^1^H} NMR spectrum at δ_P_ = −32.9 ppm, a slightly lower frequency from its dichloride **4** analogue, perhaps due to the improved π-donation by the oxygen donors. Complexes [ReO(ox-O,O)(NP_2_^Ph^O^Ar^)] (**7**) and [ReO(gly-O,O)(NP_2_^Ph^O^Ar^)] (**8**) were also synthesized by the reaction between **4** and corresponding bidentate oxalate or ethylene glycolate ligand, respectively, in the presence of a tertiary amine base with CH_2_Cl_2_ as the reaction solvent. Monitoring the course of these reactions by ^31^P{^1^H} NMR spectroscopy showed that the reaction with catechol reached completion after 24 h, oxalic acid after 36 h, while ethylene glycol took several days before appreciable amounts of the product were formed. The rate of complex formation could be increased by using MeCN and a higher temperature of 60 °C. Compared to the catecholate and oxalate analogues, **8** was relatively unstable in solution, and attempts to obtain single crystals were unsuccessful. However, the crystal structures of **6** and **7**, confirming [3 + 2] complex formation, are depicted in Figure 4.

Due to the ease of synthesis of **6**, several additional bidentate ligands, also bearing a 1,2-dihydroxyphenyl group, were added to **4** to investigate the impact of other functional groups on the formation of the [3 + 2] complexes (Scheme 2). 3,4-Dihydroxyphenyl methyl acetate and 6,7-dihydroxycoumarin were selected due to the presence of additional functionalities appended to the catechol unit. Both complexes and [ReO(cou-O,O)(NP_2_^Ph^O^Ar^)] (**9**) and [ReO(cat-O,O-COOMe)(NP_2_^Ph^O^Ar^)] (**10**) were also synthesized by the route outlined above and fully characterized. Initial attempts to use 3,4-dihydroxyphenylacetic acid as a bidentate ligand were unsuccessful, potentially due to the competitive coordination behavior of the free acid group.

### One-Pot Reactions from Re(V) Precursors

Further [3 + 2] Re-ar-X,O-NP_2_O complexes (X = O or S) could also be synthesized via one-pot reactions from the [ReOCl_3_(PPh_3_)_2_] and (NBu_4_)[ReOCl_4_] precursors and showed improved yields and purification procedures compared to the two-step method. Notably, this also indicates a preferential formation of the completed [3 + 2] ligand set relative to any intermediate complexes containing monodentate ligands such as chloride or triphenylphosphine. Homocomplexes, which would result from coordination of multiple ligands of the same type, were also not observed in these reactions.^44^

**Scheme 3:**
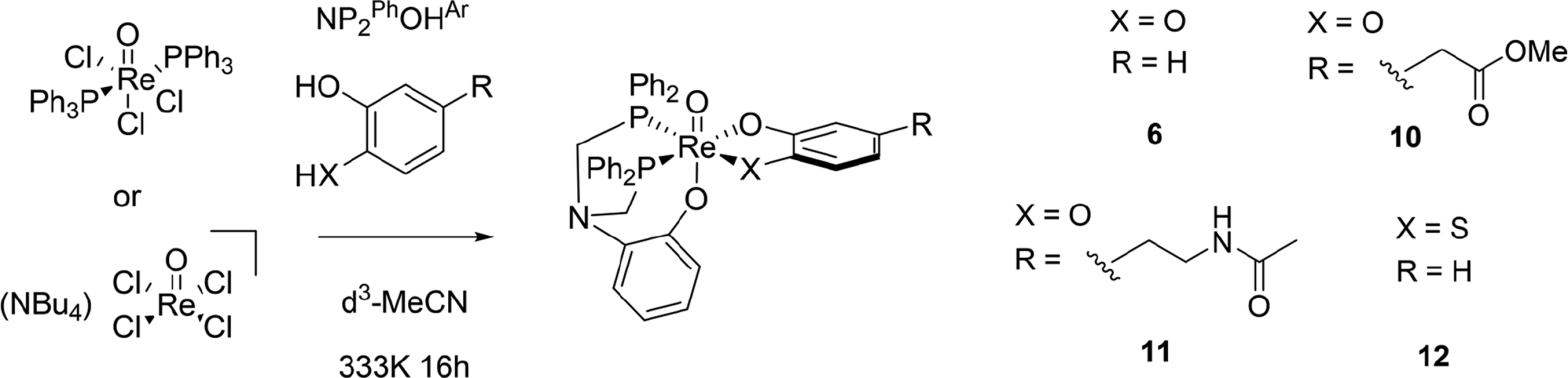
Synthesis of Example [3 + 2] Complexes from Re(V) Precursors with NP_2_^Ph^OH^Ar^ and Bidentate Oxygen-Donor Ligands under One-Pot Conditions, Including the Novel Complexes [ReO(dop-O,O)(NP_2_^Ph^O^Ar^)] (**11**) and [ReO(ar-S,O)(NP_2_^Ph^O^Ar^)] (**12**) Which Were Only Accessible by This Route

The success of a one-pot approach led us to explore a final catechol derivative with bioconjugate relevance. The structural similarity of such ligands to dopamine derivatives was noted, and consequently *N*-acetyl dopamine was synthesized readily from its free amine counterpart. Such derivatives could be coordinated to the [Re^V^O(NP_2_O)]^2+^ unit under one-pot conditions very readily. Use of the free amine was avoided to prevent any competitive coordination behavior and to improve ease of purification. The resultant complex [ReO(dop-O,O)(NP_2_^Ph^O^Ar^)] (**11**) was fully characterized by ^1^H NMR, ^31^P{^1^H} NMR, and HR-MS and further illustrates the compatibility of additional functionality on the 1,2-dihydroxyphenyl unit chelating unit (Scheme 3).

One-pot reactions also enabled access to novel [3 + 2] complexes that were otherwise difficult to obtain with a two-step reaction procedure. The complex [ReO(ar-S,O)(NP_2_^Ph^O^Ar^)] (**12**), obtained through this route, contains a coordinated 2-hydroxythiophenol ligand and is to our knowledge the first example of a {Re^V^O}^3+^ metal center stabilized by this unique combination of donor atoms: [P,P,O]/[S,O]. Due to the breaking of the *C_s_* symmetry of the [3 + 2] complex by the coordination of an asymmetric 2-hydroxythiophenol ligand, in which sulfur has replaced oxygen in the coordination sphere of the metal, the phosphorus donors are no longer chemically equivalent and a pair of resonances are seen in the ^31^P{^1^H} NMR spectrum. Such a pair of doublets were observed with a ^2^J_PP_ coupling constant value of ^2^J_PP_ = 15.0 Hz, which could be used to estimate the ^2^J_PP_ coupling constant used in the NMR simulations for the *C_s_*-symmetric {Re^V^O}^3+^ complexes.

### ^99^Tc Coordination Chemistry

The ^99^Tc analogue of the complex **3**, [^99^Tc]-[TcOCl_3_(κ^2^-NP_2_^Ph^OH^Ar^)] (**Tc-3**), was synthesized by the reaction between (NBu_4_)[^99^Tc]-[TcOCl_4_] and NP_2_^Ph^OH^Ar^ in d^3^-MeCN at RT in the absence of a base. The related bis(triphenylphosphine) complex [TcOCl_3_(PPh_3_)_2_] is unknown,^46^ and its use precluded in the synthesis of **Tc-3**, despite the favorable reactivity of its Re analogue toward the ligands described above. Nevertheless, the reaction using (NBu_4_)[TcOCl_4_] proceeded relatively cleanly with **Tc-3** formed as the major product.

**Figure 5:**
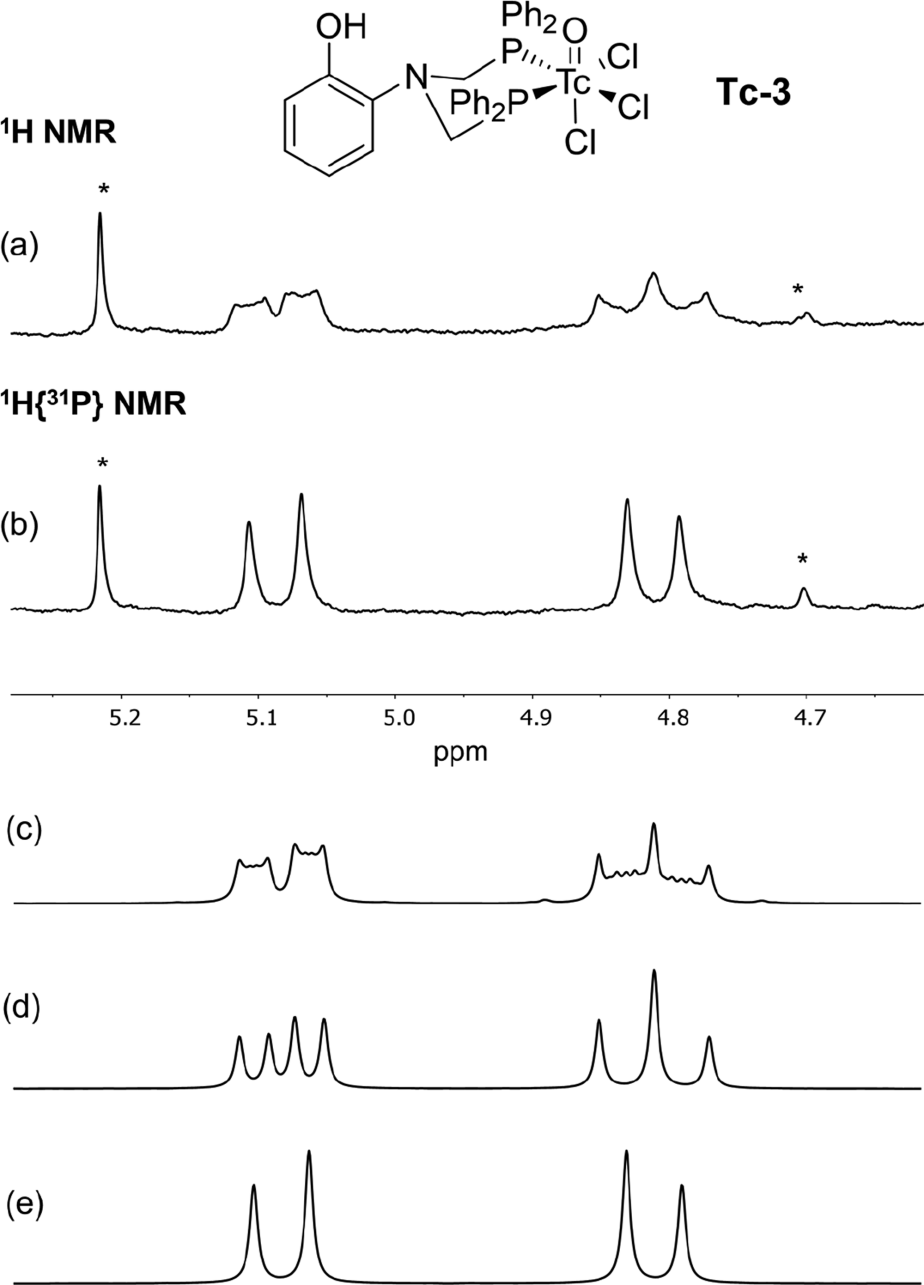
(a) ^1^H and (b) ^1^H{^31^P} NMR spectra in d^3^-MeCN of the methylene region of **Tc-3** (5.38–4.62 ppm). Full NMR spectra can be found in the SI. (c) Simulated lineshape of the ^1^H spectrum in (a). Parameters used in the simulation were ^2^J_HH′_ = 15.2 Hz; ^2^J_HP_ = 8 Hz; ^2^J_H′P_ = 15 Hz; ^2^J_PP′_ = 20 Hz; ω_1_ = 200 Hz; ω_2_ = 301 Hz; lb = 8 Hz. (d) Same parameters as in panel (c) but with ^2^J_PP′_ = 0 Hz. (e) Same parameters as in panel (c) but with ^31^P decoupling ^2^J_HP_ = ^2^J_H′P_ = 0 Hz. Asterisks indicate solvent impurities not associated with the spin system.

**Tc-3** bears a strong structural similarity to **3**, as evidenced by the highly similar coupling pattern observed for the methylene protons in its ^1^H NMR spectrum in d^3^-MeCN. Figure 5 depicts the region of the ^1^H NMR and ^1^H{^31^P} spectra for **Tc-3** corresponding to the methylene resonances. These spectra are suggestive of a AA’BB’XX’ spin system in which highly similar lineshapes to that of the Re analogue are observed for the bidentate coordination mode of the ligand. Upon broadband ^31^P decoupling, only the ^2^J_HH′_ geminal coupling between the methylene hydrogens in **Tc-3** is observed with a value of 15.2 Hz (compared to 15.9 Hz for **3**). The presence of these lineshapes in the ^1^H NMR spectrum is strongly indicative of a bidentate coordinated phosphine ligand. The ^31^P{^1^H} spectrum for **Tc-3** contains a lone singlet at +27.5 ppm, which is considerably higher frequency relative to the analogous Re complex **3**. Considering the similar chemical shifts observed for the methylene hydrogens in **3** and **Tc-3**, this large shift to higher frequency likely results from inherent differences between Tc(V) and Re(V), rather than any significant structural dissimilarity.

A second species was formed as a minor product in the same reaction. The ^1^H NMR spectrum indicated the presence of this species by the presence of a singlet resonance in the methylene region of the spectrum at δ_H_ = +4.28 ppm, and by analogy to the Re case, this can ascribed to the presence of a {Tc^V^O_2_}^+^ species analogous to **5**, [TcO_2_Cl(κ^2^-NP_2_^Ph^OH^Ar^)] (**Tc-5**). Such a species is not expected to exhibit a complicated lineshape corresponding to an AA’BB’XX’ spin system due to the chemical equivalence of the methylene hydrogens. A singlet at δ_P_ = +26.9 ppm is observed in the ^31^P{^1^H} NMR spectrum for **Tc-5**. Considering that formation of this species is observed even using a room temperature synthesis, it suggests that the hydrolysis pathway to {M^V^O_2_}^+^ from {M^V^O}^3+^ for Tc in such systems is more facile than in the case of Re, as expected from the rates of substitution reactions on 2nd vs 3rd row transition metals.^90^ Indeed, if elevated temperatures are employed or a base is added to the reaction mixture for this synthesis, in order to promote the formation of a *tridentate* complex, analogous to **4**, then no such species is observed but rather the relative amounts of **Tc-3** and **Tc-5** are altered to favor the latter. This is consistent with the behavior of Re(V) derivatives in which hydrolysis of **3** to **5** is promoted in the presence of base when sufficient water is present in the system. Using anhydrous solvents without base favors the formation of **Tc-3**.

**Figure 6:**
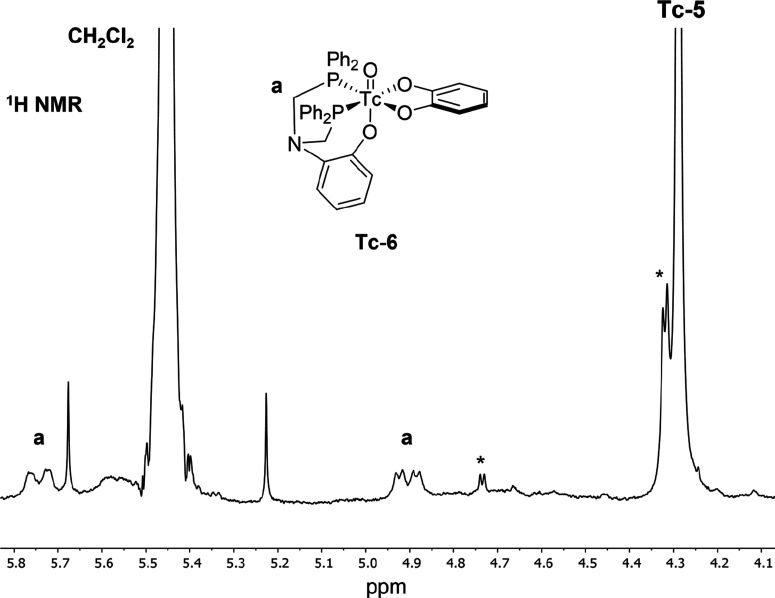
^1^H NMR spectrum (d^3^-MeCN, 500 MHz, 298 K) for the 5.8–4.1 ppm region obtained for the attempted one-pot synthesis of **Tc-6**. The peaks labeled (a) correspond to the methylene hydrogens of the coordinated ligand. Asterisks indicate unidentified impurities.

It is possible that the presence of a stabilizing bidentate catechol or other dioxygen ligand could favor the formation of a [3 + 2] Tc(V) complex in preference to the hydrolyzed dioxo species such as **Tc-5**. Consequently, a one-pot synthetic procedure was attempted using catechol. Although this reaction resulted in the formation of **Tc-5** as the major product, the presence of another Tc-containing species observed in the ^1^H NMR, indicated by the presence of a distinctive methylene region lineshape. Figure 6 depicts the methylene region obtained for this reaction. This lineshape is comparable to that observed for the Re derivative **6**.

Thin-layer chromatography analysis of this mixture in 5% MeOH:CH_2_Cl_2_ alongside a pure sample of **6** facilitated chromatographic comparison between the components of the Tc reaction and a pure [3 + 2] complex. A yellow band was observed with *R*_f_ = 1.0 in both cases (see the SI), strongly suggesting the presence of isostructural **Tc-6** in the crude reaction mixture. Additionally, low-resolution mass spectrometry also indicated the presence of **Tc-6** through observation of a signal at *m*/*z* = 728.1 (calc. [M + H] at *m*/*z* = 728.1).

Unfortunately, attempts to isolate **Tc-6** by silica column chromatography resulted in further decomposition of the product, and appreciable material for further analysis could not be recovered. It is likely that the hydrolysis pathway necessitates rigorous conditions to promote formation of **Tc-6** during reaction and purification, if stable [3 + 2] complexes derived from Tc(V) are to be isolated.

## Conclusions

The present study demonstrates that N-centered phosphine ligands can be used to prepare air and moisture-stable {Re^V^O}^3+^ complexes containing bidentate and tridentate coordination modes. A tridentate [ReOCl_2_(NP_2_^Ph^O^Ar^)] complex can function as a reactive “metal-fragment” toward further functionalization with oxygen (and sulfur) donors to form [3 + 2] mixed-ligand sets. Control over bidentate and tridentate coordination can be achieved through the choice of third arm donor of the ligand and the presence of base in the reaction medium. A distinctive AA’BB’XX’ spin system formed through *cis* coordination of the diphosphine to the metal center aids rapid diagnosis of the binding mode through a large change in lineshapes for the ^1^H NMR resonances corresponding to the bridging methylene groups of the ligand. These lineshapes have been successfully recreated by NMR simulation for a 6-spin system, with the magnitude of ^2^J_PH_ coupling constants being a critical parameter in their appearance and highly second-order lineshapes arising due to the passive ^2^J_PP′_ coupling mediated via the metal center.

The successful formation of [3 + 2] complexes under one- or two-step reaction conditions from Re(V) oxo precursors has been shown to be compatible with a range of functionalities on the bidentate unit. The functionalities selected in this study represent a useful starting point from which further targeting units could be appended to [ReO(NP_2_^Ph^O^Ar^)]^2+^ units with well-defined coordination spheres. Further work in our group is looking at the coordination chemistry of cysteine residues with this “metal-fragment”.

The extension of such ligand systems to the formation of [3 + 2] complexes with {Tc^V^O}^3+^ is complicated by hydrolysis to {Tc^V^O_2_}^+^ units under a range of conditions. It is anticipated that this may be improved through further study toward the selection of a bidentate ligand with a rapid tendency to coordinate to {Tc^V^O}^3+^ units and stabilize a [3 + 2] mixed-ligand set.

Further studies in our group are looking at the extension of NP_2_^Ph^X^R^ ligands to the formulation of mixed-ligand [3 + 2] complexes based on mono-imido {M^V^(NR)}^3+^ (M = Re, ^99^Tc) cores, with the potential for greater stability toward hydrolysis. Likewise, the recurrent appearance of the {M^V^O_2_}^+^ core in our synthetic procedures has led us to consider the use of this related core in the formulation of heterofunctionalized bis(diphosphine) complexes toward targeted nuclear imaging.

## Supporting Information Available

Tables containing structural parameters and ellipsoidal representations of crystal structures, NMR spectra for metal complexes, ^99^Tc TLC data and details of ligand synthesis (PDF)

## Accession Codes

CCDC 2112628–2112631 and 2129875 contain the supplementary crystallographic data for this paper. These data can be obtained free of charge via www.ccdc.cam.ac.uk/data_request/cif, or by emailing data_request@ccdc.cam.ac.uk, or by contacting The Cambridge Crystallographic Data Centre, 12 Union Road, Cambridge CB2 1EZ, UK; fax: +44 1223 336033.
