# Supplementary material for: N-Centered Tripodal Phosphine Re(V) and Tc(V) Oxo Complexes: Revisiting a [3 + 2] Mixed-Ligand Approach
Source: Inorg Chem. 2022 May 11;61(20):8000–14. doi: 10.1021/acs.inorgchem.2c00693 (PMC9131457; doi:10.1021/acs.inorgchem.2c00693)
Supplement: Supplementary file 1 — ic2c00693_si_001.pdf [file ic2c00693_si_001.pdf]

Supporting Information

N-centered Tripodal Phosphine Re(V) and Tc(V) Oxo Complexes: Revisiting a [3+2] Mixed-ligand Approach

Saul M. Cooper,[1,2] Andrew J.P. White,[1] Thomas R. Eykyn,[2] Michelle T. Ma,[2] Philip W. Miller,[1*] Nicholas J. Long[1*]

1) Department of Chemistry, Imperial College London, Molecular Sciences Research Hub, 82 Wood Lane, White City Campus, London, W12 0BZ, UK.

2) School of Biomedical Engineering & Imaging Sciences, King's College London, 4th Floor Lambeth Wing, St Thomas' Hospital, London, SE1 7EH, UK.

*Corresponding Authors: Nicholas J. Long (n.long@imperial.ac.uk)
Philip W. Miller (philip.miller@imperial.ac.uk)

1) X-Ray Crystallography

Table S1. Crystal Data, Data Collection and Refinement Parameters for the structures of **2**, **3**, **4**, **6** and **7**.

data	2	3	4
**formula**	$C_{39}H_{36}Cl_3NO_2P_3Re$	$C_{32}H_{29}Cl_3NO_2P_2Re$	$C_{32}H_{28}Cl_2NO_2P_2Re$
**solvent**	CH_2Cl_2	C_3H_6O	—
**formula weight**	1021.07	872.13	777.59
**colour, habit**	blue tablets	brown blocky needles	brown blocks
**temperature / K**	173	173	173
**crystal system**	orthorhombic	monoclinic	monoclinic
**space group**	$Pna2_1$ (no. 33)	$P2_1/n$ (no. 14)	$P2_1$ (no. 4)
a **/ Å**	16.3590(5)	10.0503(3)	9.8109(2)
b **/ Å**	24.6439(9)	10.3341(4)	15.8857(3)
c **/ Å**	9.9761(3)	32.8841(12)	9.8228(2)
**α / deg**	90	90	90
**β / deg**	90	90.487(3)	105.895(2)
**γ / deg**	90	90	90
V **/ Å**^{3}	4021.9(2)	3415.2(2)	1472.40(6)
Z	4	4	2
D_c **/ g cm**$^{-3}$	1.686	1.696	1.754
**radiation used**	Mo-Kα	Mo-Kα	Mo-Kα
**μ / mm**$^{-1}$	3.510	3.923	4.448
**no. of unique reflns**			
**measured (**R_{int}**)**	5976 (0.0234)	6896 (0.0284)	4759 (0.0211)
**obs,** $\lvert F_o\rvert > 4\sigma(\lvert F_o\rvert)$	5076	5989	4630
**completeness (%)** [a]	99.1	99.1	99.8
**no. of variables**	497	427	361
R_1**(obs),** wR_2**(all)** [b]	0.0279, 0.0564	0.0413, 0.0698	0.0207, 0.0460

[a] Completeness to 0.84 Å resolution. [b] $R_1 = \Sigma||F_o| - |F_c||/\Sigma|F_o|$; $wR_2 = \{\Sigma[w(F_o^2 - F_c^2)^2] / \Sigma[w(F_o^2)^2]\}^{1/2}$; $w^{-1} = \sigma^2(F_o^2) + (aP)^2 + bP$.

Table S1. …continued

data	6	7
**formula**	$C_{38}H_{32}NO_4P_2Re$	$C_{34}H_{28}NO_6P_2Re$
**solvent**	1.5(CH_2Cl_2)	CH_2Cl_2
**formula weight**	942.17	879.64
**colour, habit**	dark brown blocks	red tablets
**temperature / K**	173	173
**crystal system**	cubic	monoclinic
**space group**	*Ia*–3 (no. 206)	$P2_1$ (no. 4)
a **/ Å**	37.1248(3)	9.39968(11)
b **/ Å**	37.1248(3)	18.2280(2)
c **/ Å**	37.1248(3)	9.93251(11)
**α / deg**	90	90
**β / deg**	90	98.6575(10)
**γ / deg**	90	90
V **/** $Å^3$	51167.4(12)	1682.42(3)
Z	48	2
D_c **/ g** cm^{-3}	1.468	1.736
**radiation used**	Mo-Kα	Mo-Kα
**μ /** mm^{-1}	3.150	3.912
**no. of unique reflns**		
**measured (**R_{int}**)**	8709 (0.0259)	7668 (0.0401)
**obs,** $\lvert F_o \rvert > 4\sigma(\lvert F_o \rvert)$	6515	7396
**completeness (%)** [a]	98.9	99.9
**no. of variables**	416	438
R_1**(obs),** wR_2**(all)** [b]	0.0392, 0.1036	0.0190, 0.0374

Table S1 provides a summary of the crystallographic data for the structures of **2**, **3**, **4**, **6** and **7**. Data were collected using an Agilent Xcalibur 3 E diffractometer, and the structures were refined using the SHELXTL and SHELX-2013 program systems.[1, 2] The absolute structures of **2**, **4** and **7** were determined by use of the Flack parameter [x^+ = –0.027(7), –0.024(5) and –0.020(2) respectively]. CCDC 2112628 to 2112631 for **2**, **3**, **4** and **6** respectively, and 2129875 for **7**.

The C50-based included dichloromethane solvent molecule in the structure of **2** was found to be disordered. Three orientations were identified of *ca*. 79, 11 and 10% occupancy, their geometries were optimised, the thermal parameters of adjacent atoms were restrained to be similar, and only the non-hydrogen atoms of the major occupancy orientation were refined anisotropically (those of the minor occupancy orientations were refined isotropically). The C40-based included acetone solvent

molecule in the structure of **3** was found to be disordered. Two orientations were identified of *ca*. 56 and 44% occupancy, their geometries were optimised, the thermal parameters of adjacent atoms were restrained to be similar, and only the non-hydrogen atoms of the major occupancy orientation were refined anisotropically (those of the minor occupancy orientation were refined isotropically). The O12–H hydrogen atom in the structure of **3** was located from a ΔF map and refined freely subject to an O–H distance constraint of 0.90 Å. The C31-based phenyl ring in the structure of **6** was found to be disordered. Two orientations were identified of *ca*. 79 and 21% occupancy, their geometries were optimised, the thermal parameters of adjacent atoms were restrained to be similar, and only the non-hydrogen atoms of the major occupancy orientation were refined anisotropically (those of the minor occupancy orientation were refined isotropically). The included solvent in the structure of **6** was found to be highly disordered, and the best approach to handling this diffuse electron density was found to be the SQUEEZE routine of PLATON.[3] This suggested a total of 2979 electrons per unit cell, equivalent to 62.1 electrons per asymmetric unit. Before the use of SQUEEZE the solvent present could not be clearly distinguished, and so the most recently used solvent dichloromethane (CH_2Cl_2, 42 electrons) was assumed, and 1.5 dichloromethane molecules corresponds to 63 electrons so this was used as the solvent present. As a result, the atom list for the asymmetric unit is low by 1.5(CH_2Cl_2) = $C_{1.5}H_3Cl_3$ (and that for the unit cell low by $C_{72}H_{144}Cl_{144}$) compared to what is actually presumed to be present. The C50-based included dichloromethane solvent molecule in the structure of **7** was found to be disordered. Two orientations were identified of *ca*. 82 and 18% occupancy, their geometries were optimised, the thermal parameters of adjacent atoms were restrained to be similar, and only the non-hydrogen atoms of the major occupancy orientation were refined anisotropically (those of the minor occupancy orientation were refined isotropically).

Figures

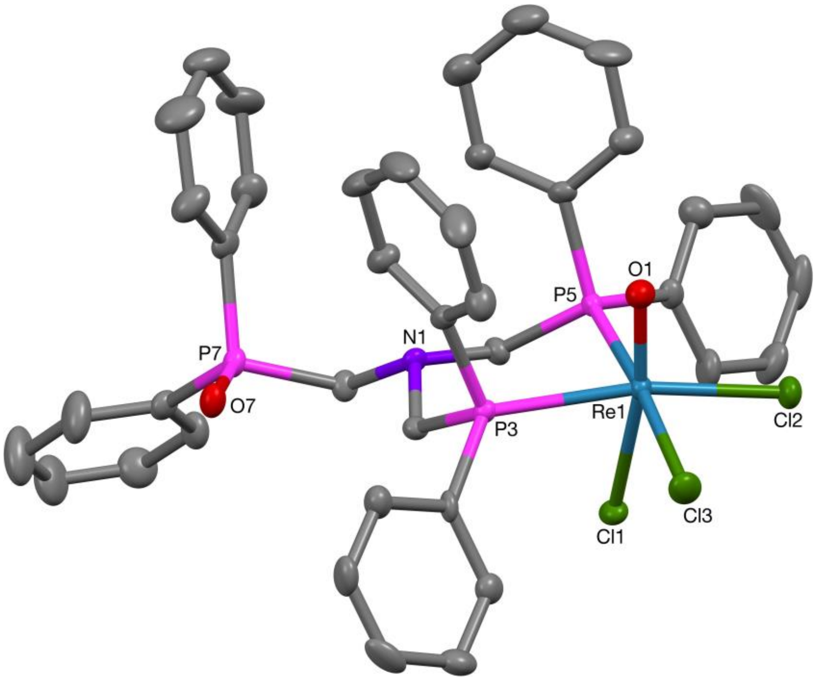

Figure S1 The crystal structure of **2** (50% probability ellipsoids).

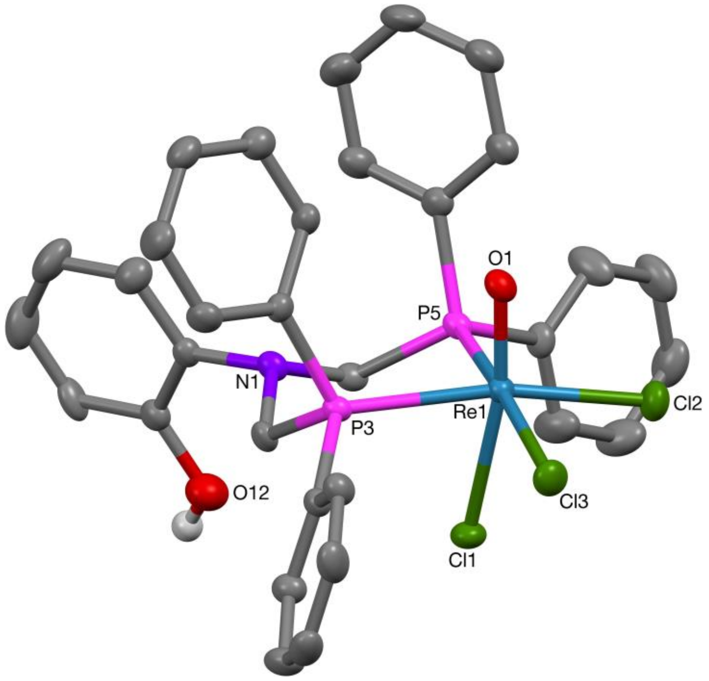

Figure S2 The crystal structure of **3** (50% probability ellipsoids).

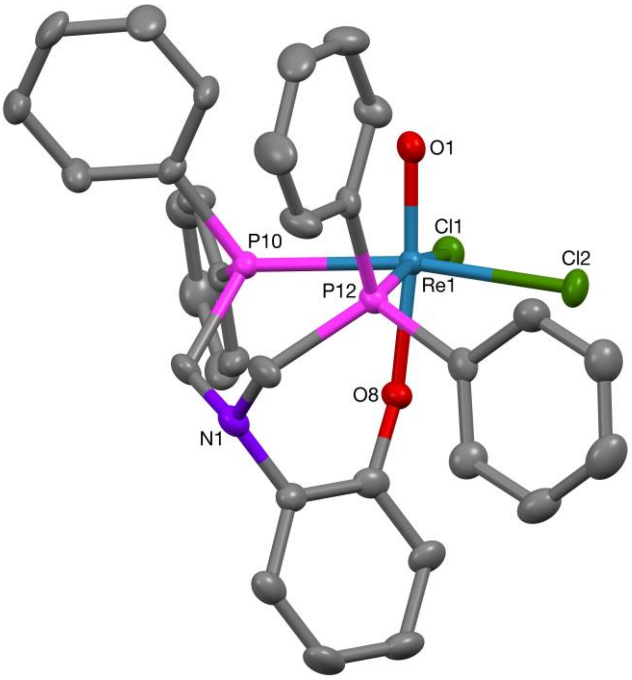

Figure S3 The crystal structure of **4** (50% probability ellipsoids).

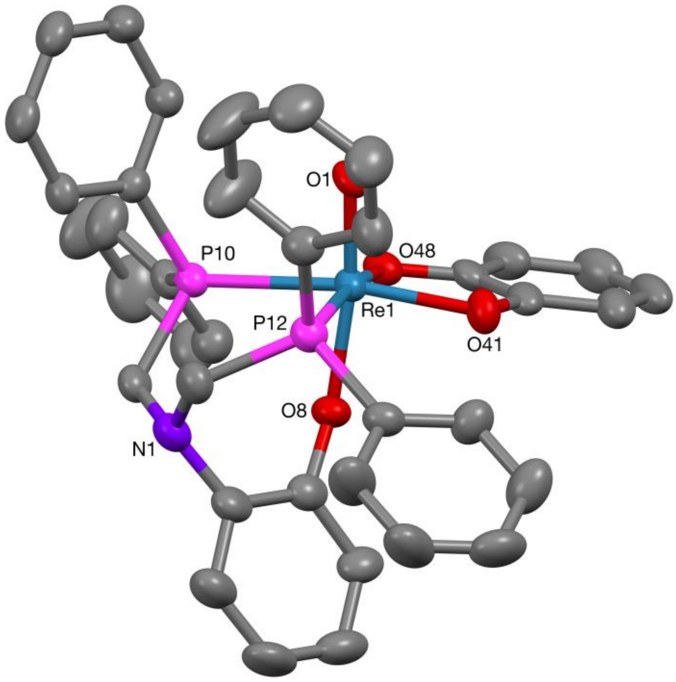

Figure S4 The crystal structure of **6** (50% probability ellipsoids).

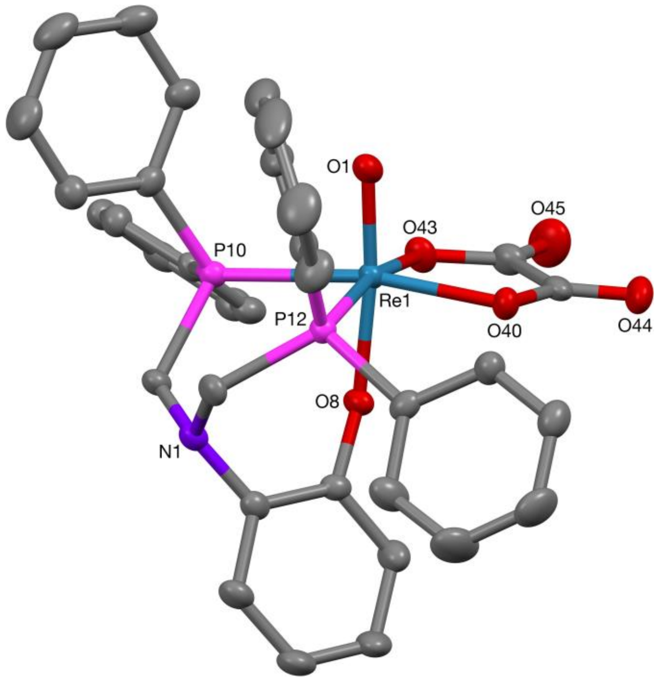

Figure S5 The crystal structure of **7** (50% probability ellipsoids).

2) NMR Spectra for $\{Re^{V}O\}^{3+}$ and $\{^{99}Tc^{V}O\}^{3+}$ Complexes

Figure S6: $^{31}P\{^{1}H\}$ NMR Spectrum (THF, C_6D_6 capillary, 298 K, 162 MHz) of $[ReOCl_3(\kappa^2\text{-}NP_2^{Ph}P^{Ph})]$ (**Re-NP$_3$**) (**1**) (*in situ* monitoring of reaction progression).

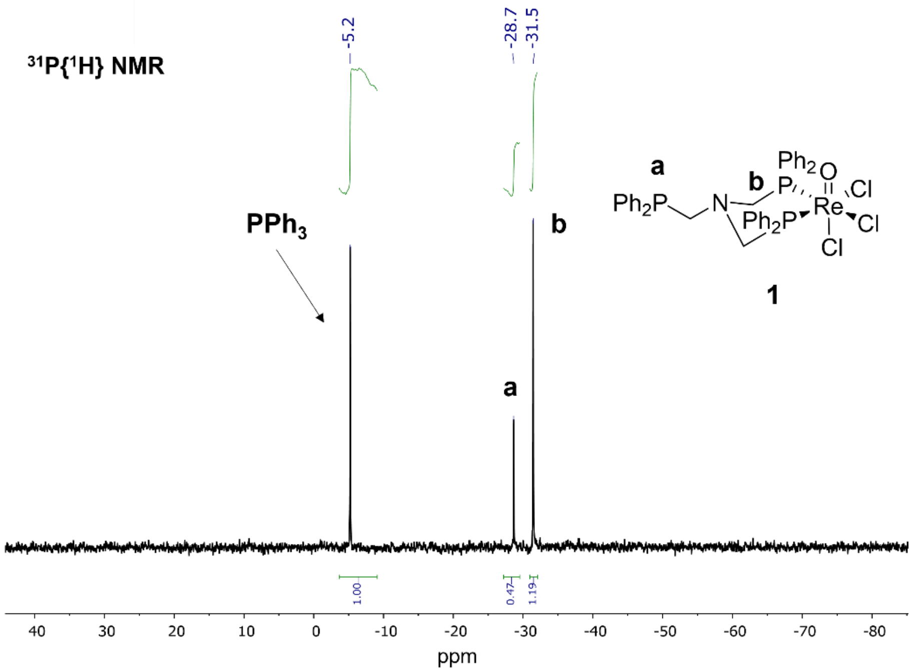

Figure S7: ^{1}H NMR Spectrum (CD_2Cl_2, 298 K, 400 MHz) of $[ReOCl_3(\kappa^2\text{-}NP_2^{Ph}P(O)^{Ph})]$ (**Re-NP$_2$PO**) (**2**)

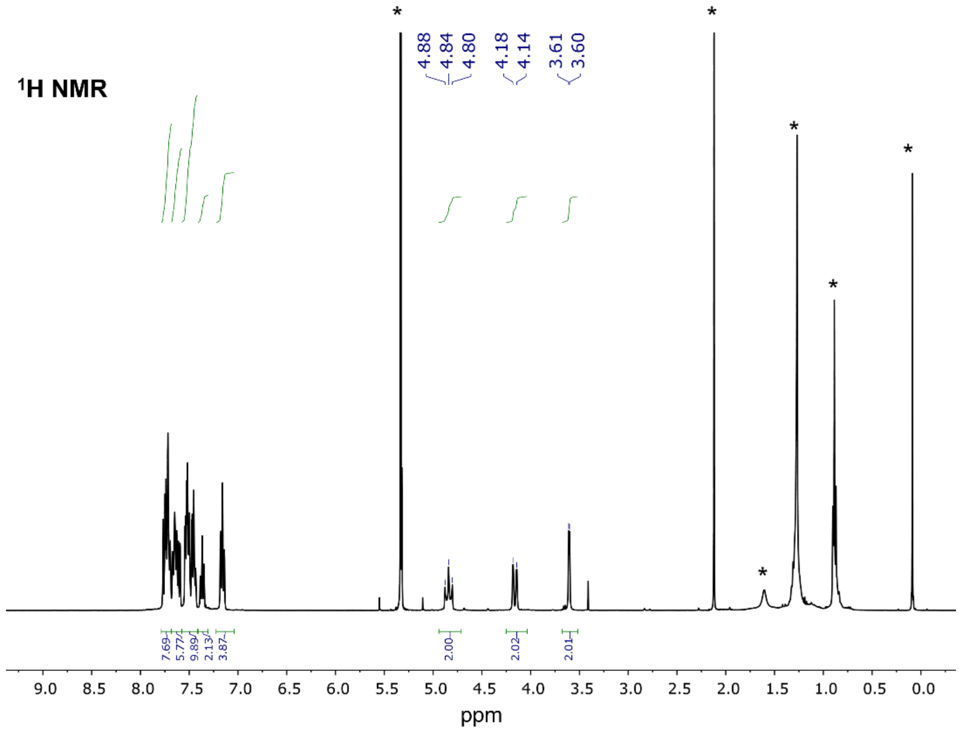

Figure S8: ^{13}C NMR Spectrum (CD_2Cl_2, 298 K, 101 MHz) of [$ReOCl_3$(κ^2-$NP_2^{Ph}P(O)^{Ph}$)] (**Re-NP₂PO**) (**2**)

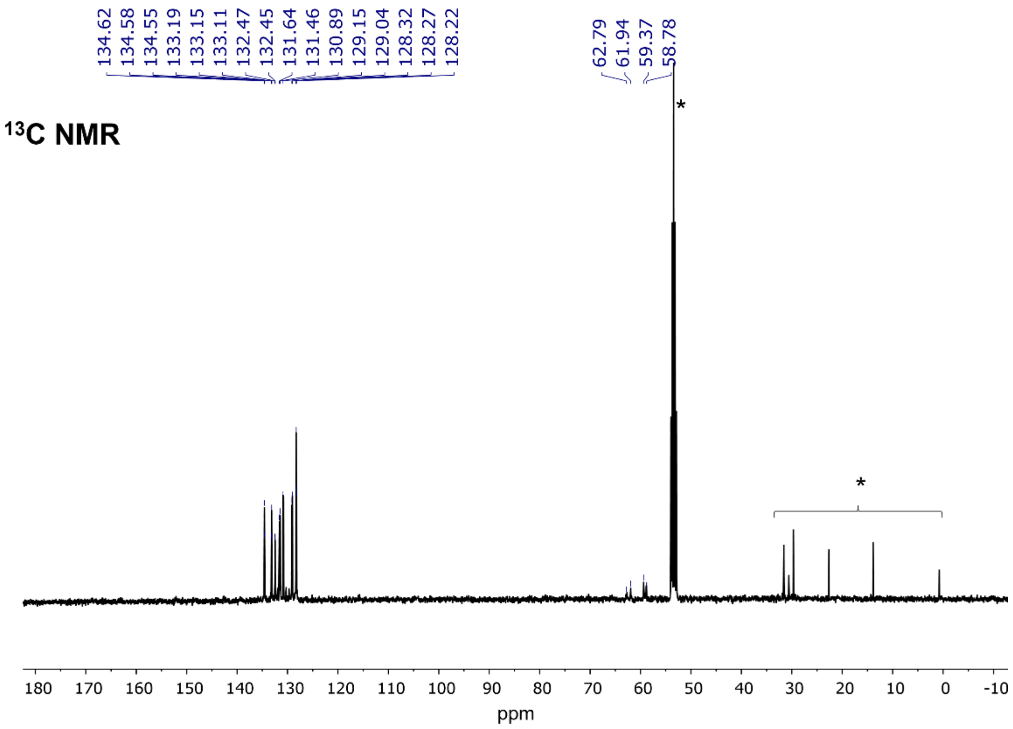

Figure S9: $^{31}P\{^1H\}$ NMR Spectrum (CD_2Cl_2, 298 K, 162 MHz) of [$ReOCl_3$(κ^2-$NP_2^{Ph}P(O)^{Ph}$)] (**Re-NP₂PO**) (**2**)

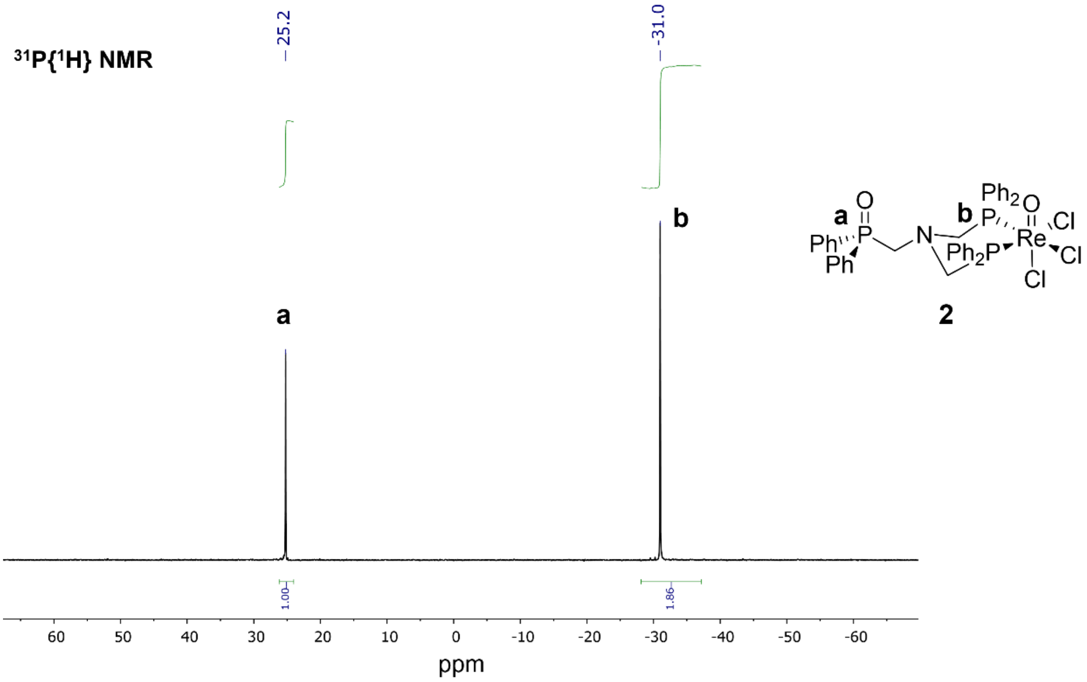

Figure S10: ^{1}H NMR Spectrum (d^3-MeCN, 298 K, 500 MHz) of [ReOCl$_3$(κ^2-NP$_2$^{Ph}OH^{Ar})] (**Re-NP$_2$OH**) (**3**)

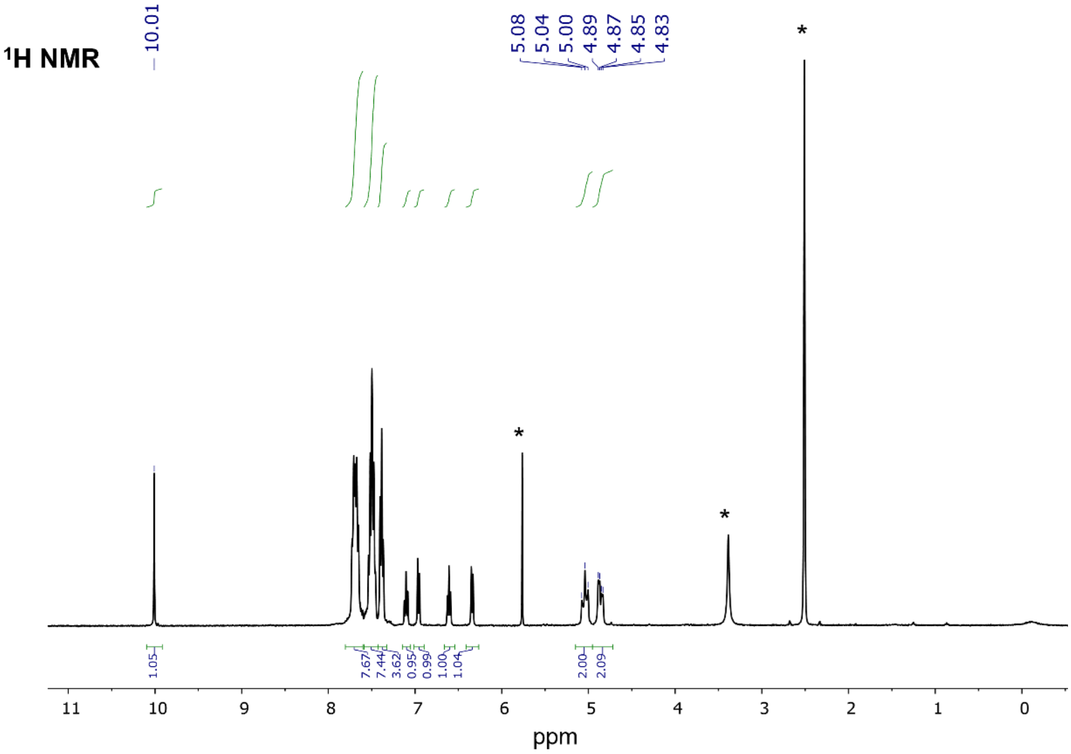

^{13}C NMR Spectrum precluded by solubility in d^3-MeCN

Figure S11: ^{31}P{^{1}H} NMR Spectrum (d^3-MeCN, 298 K, 162 MHz) of [ReOCl$_3$(κ^2-NP$_2$^{Ph}OH^{Ar})] (**Re-NP$_2$OH**) (**3**)

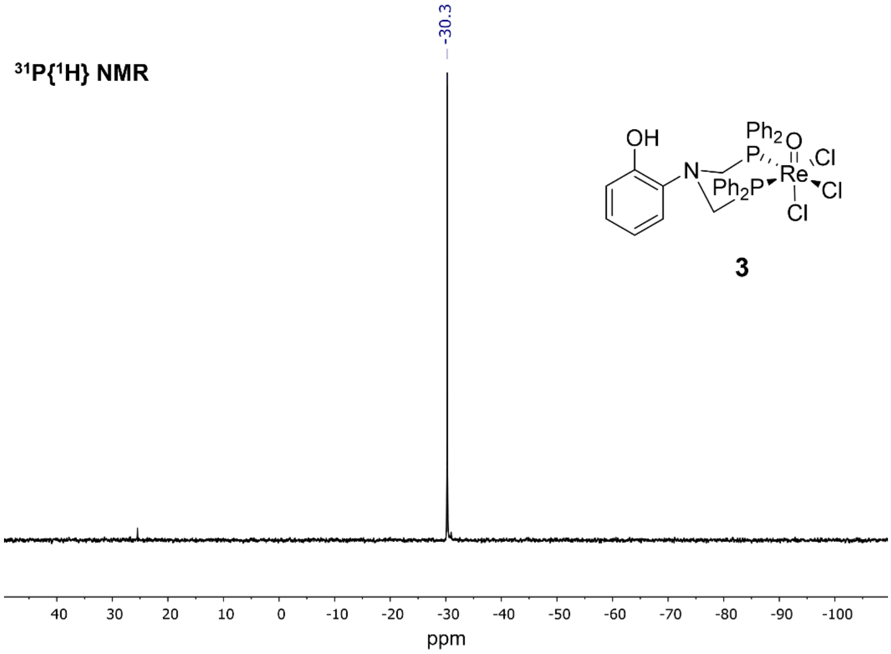

3

Figure S12: ^{1}H NMR Spectrum (d^{3}-MeCN, 298 K, 500 MHz) of [ReOCl$_2$(κ^{3}-NP$_2$^{Ph}O^{Ar})] (**Re-NP$_2$O**) (**4**)

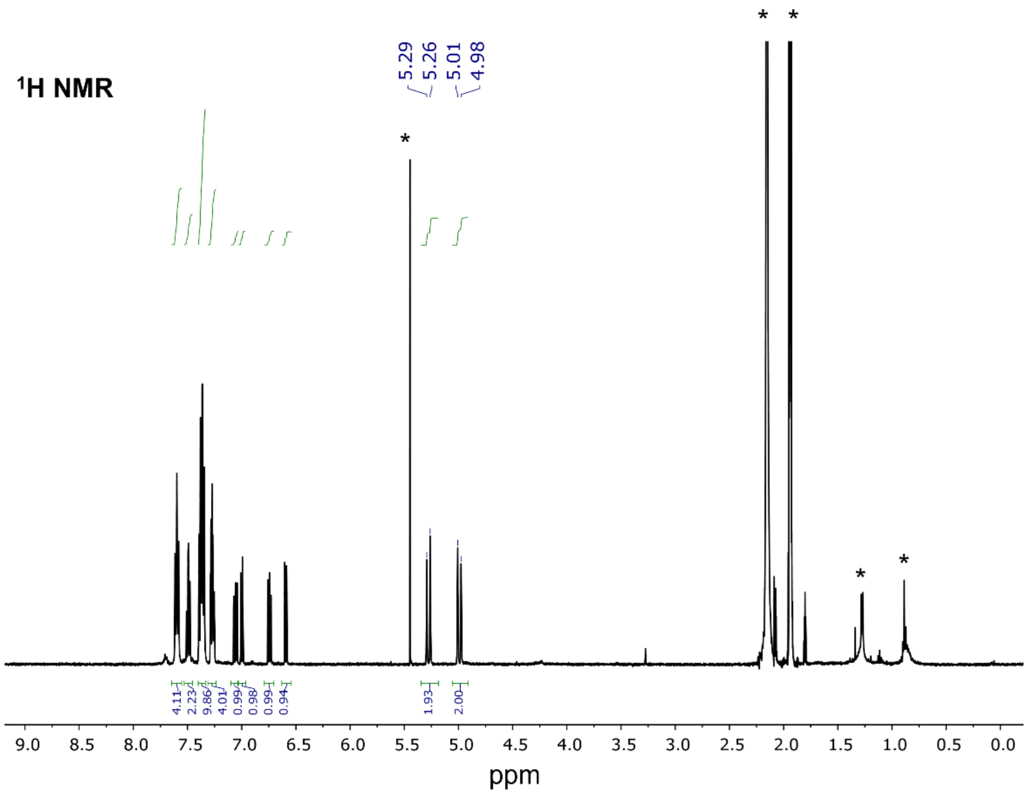

Figure S13: ^{13}C NMR Spectrum (CD_2Cl_2, 298 K, 101 MHz) of [ReOCl$_2$(κ^{3}-NP$_2$^{Ph}O^{Ar})] (**Re-NP$_2$O**) (**4**)

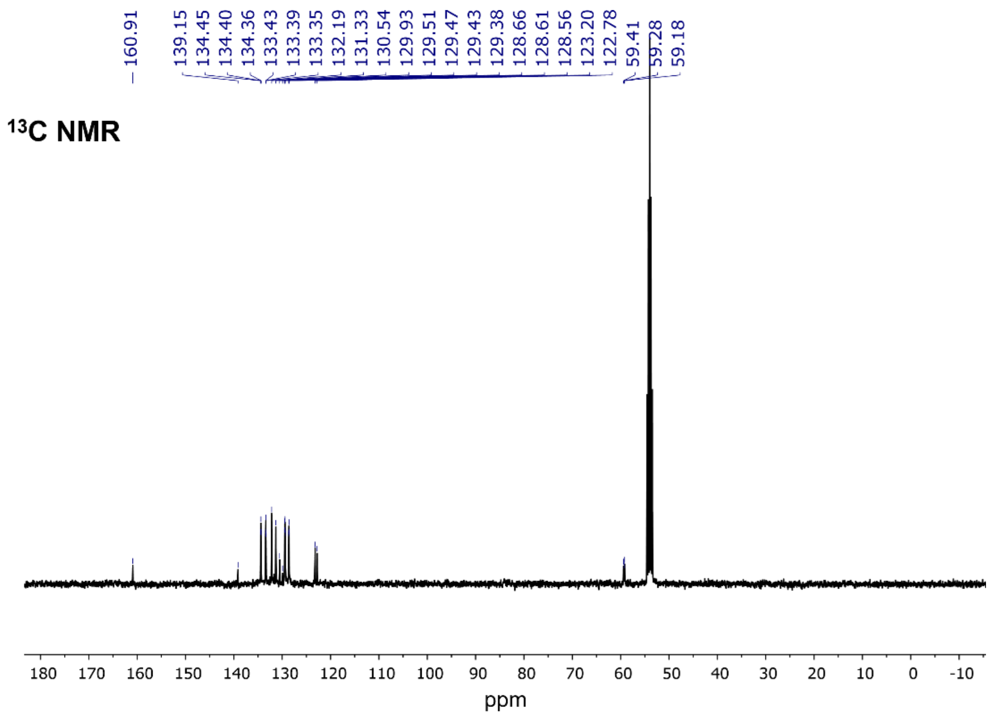

Figure S14: $^{31}P\{^{1}H\}$ NMR Spectrum (CD_2Cl_2, 298 K, 162 MHz) of [ReOCl$_2$(κ^3-NP$_2^{Ph}$O^{Ar})] (**Re-NP$_2$O**) (**4**)

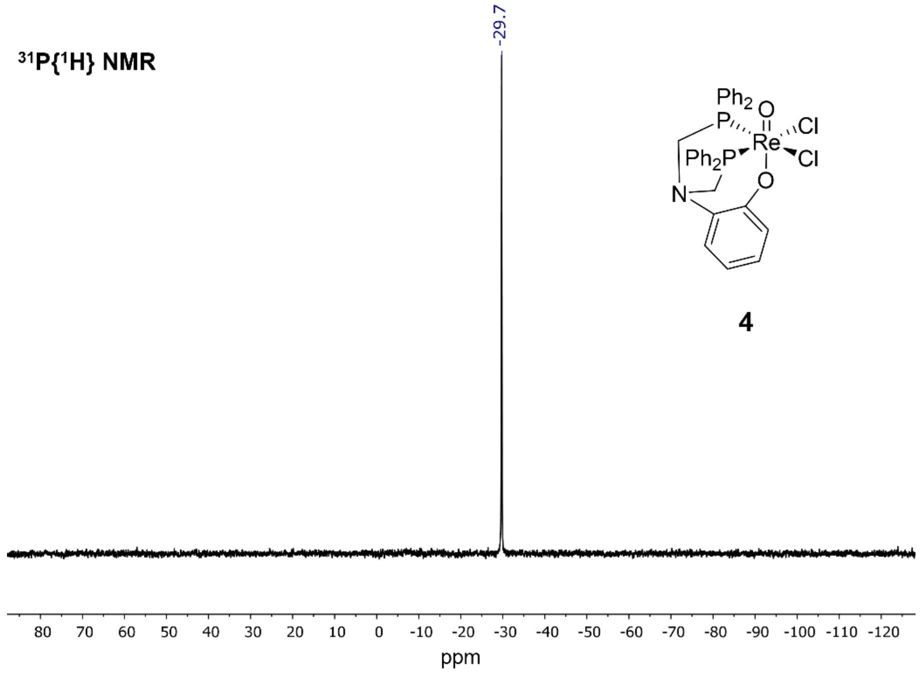

Figure S15: ^{1}H NMR Spectrum (d^3-MeOD, 298 K, 400 MHz) of [ReO$_2$Cl(κ^2-NP$_2^{Ph}$OH^{Ar})] (**ReO$_2$-NP$_2$OH**) (**5**)

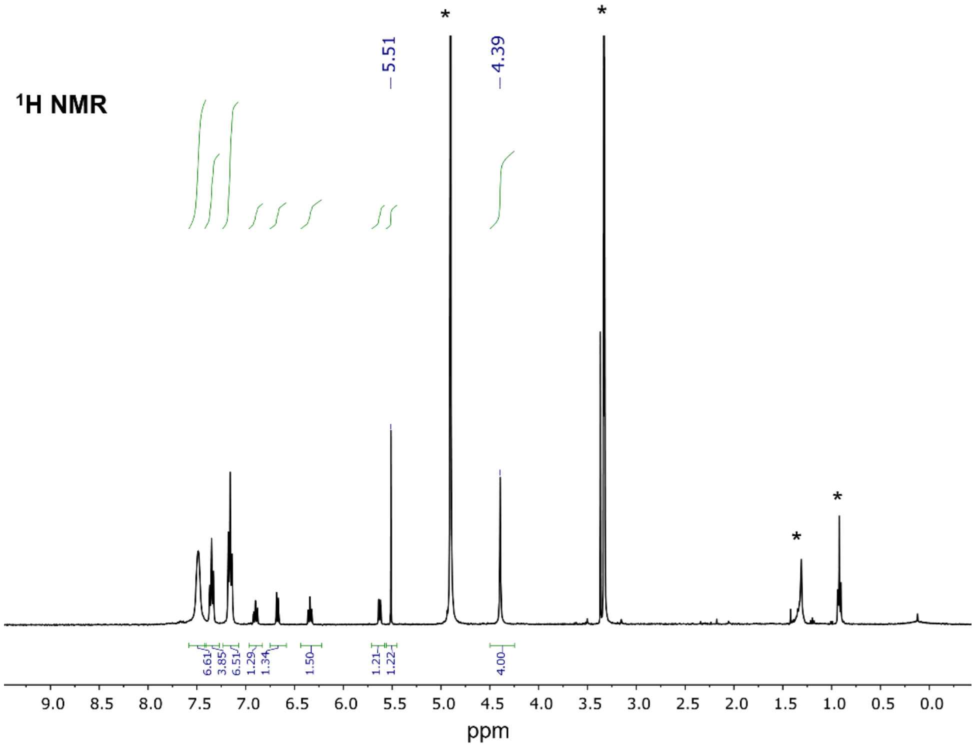

Figure S16: $^{31}P\{^1H\}$ NMR Spectrum (d^3-MeOD, 298 K, 162 MHz) of [$ReO_2Cl(\kappa^2$-$NP_2^{Ph}OH^{Ar})$] (**ReO_2-NP_2OH**) (**5**)

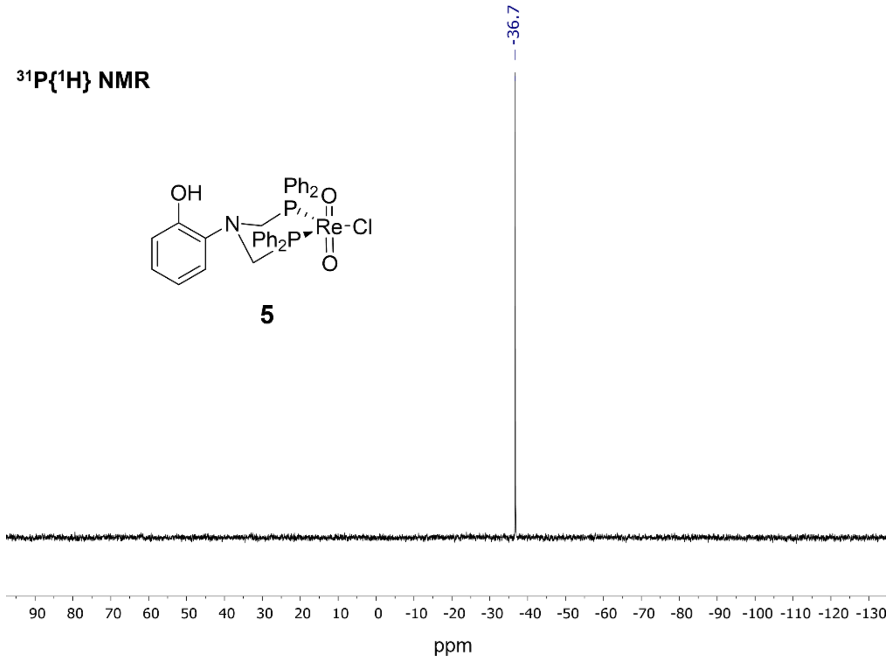

Figure S17: ^1H NMR Spectrum ($CDCl_3$, 298 K, 400 MHz) of [ReO(cat-O,O)(κ^3-$NP_2^{Ph}O^{Ar}$)] (**Re-cat-O,O-NP_2O**) (**6**)

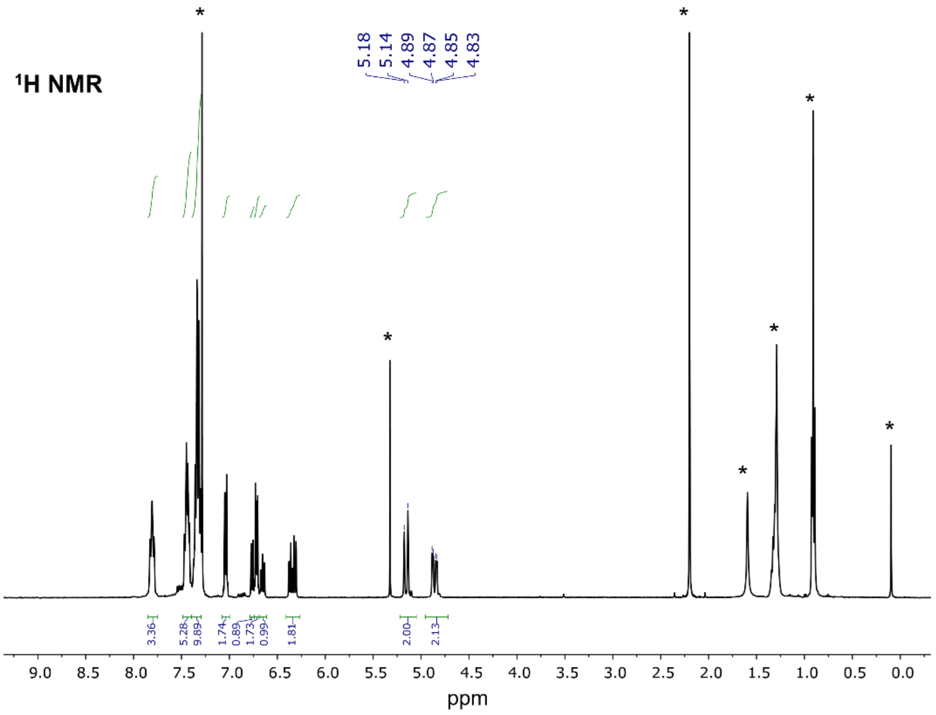

Figure S18: ^{13}C NMR Spectrum ($CDCl_3$, 298 K, 101 MHz) of [ReO(cat-O,O)(κ^3-$NP_2^{Ph}O^{Ar}$)] (**Re-cat-O,O-NP$_2$O**) (**6**)

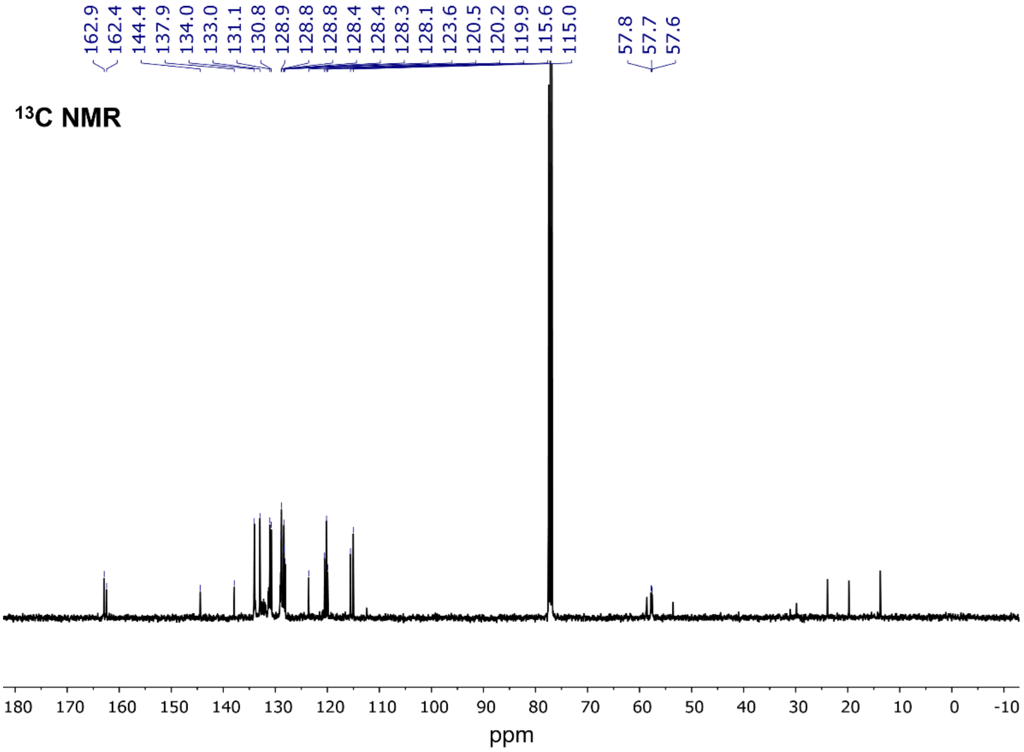

Figure S19: $^{31}P\{^1H\}$ NMR Spectrum ($CDCl_3$, 298 K, 162 MHz) of [ReO(cat-O,O)(κ^3-$NP_2^{Ph}O^{Ar}$)] (**Re-cat-O,O-NP$_2$O**) (**6**)

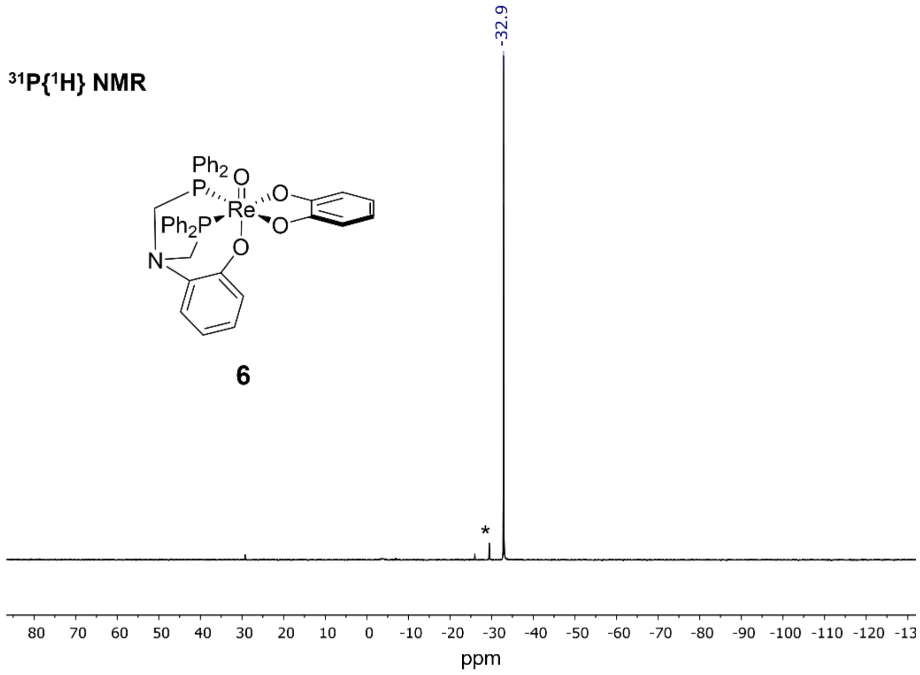

Figure S20: ^{1}H NMR Spectrum ($CDCl_3$, 298 K, 400 MHz) of [ReO(ox-O,O)(κ^3-$NP_2^{Ph}O^{Ar}$)] (**Re-ox-O,O-NP$_2$O**) (**7**)

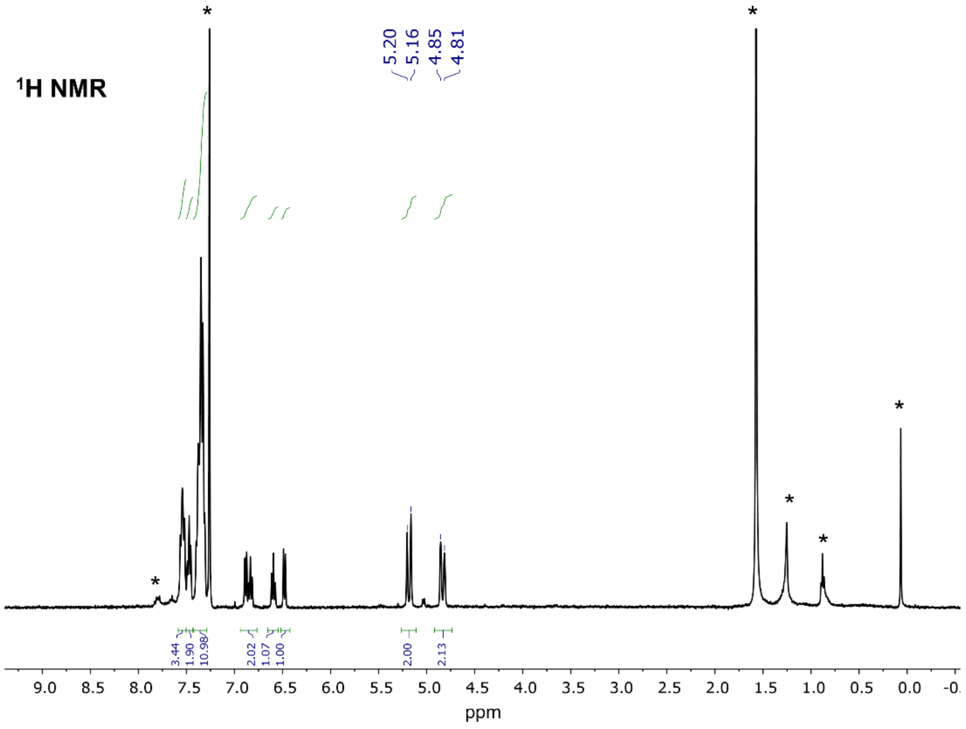

Figure S21: $^{31}P\{^{1}H\}$ NMR Spectrum ($CDCl_3$, 298 K, 162 MHz) of [ReO(ox-O,O)(κ^3-$NP_2^{Ph}O^{Ar}$)] (**Re-ox-O,O-NP$_2$O**) (**7**)

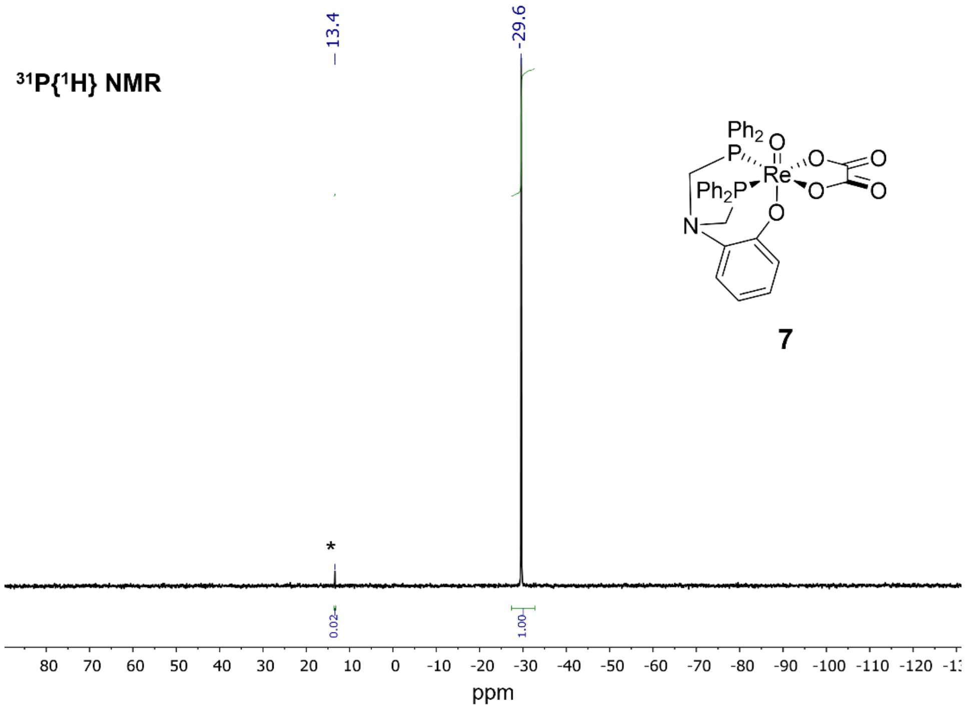

Figure S22: Partial ^1H NMR Spectrum (CD_2Cl_2, 298 K, 400 MHz) of [ReO(gly-O,O)(κ^3-$NP_2^{Ph}O^{Ar}$)] (**Re-gly-O,O-NP$_2$O**) (**8**). Spectrum below 2.5 ppm with additional solvent impurities has been omitted for clarity.

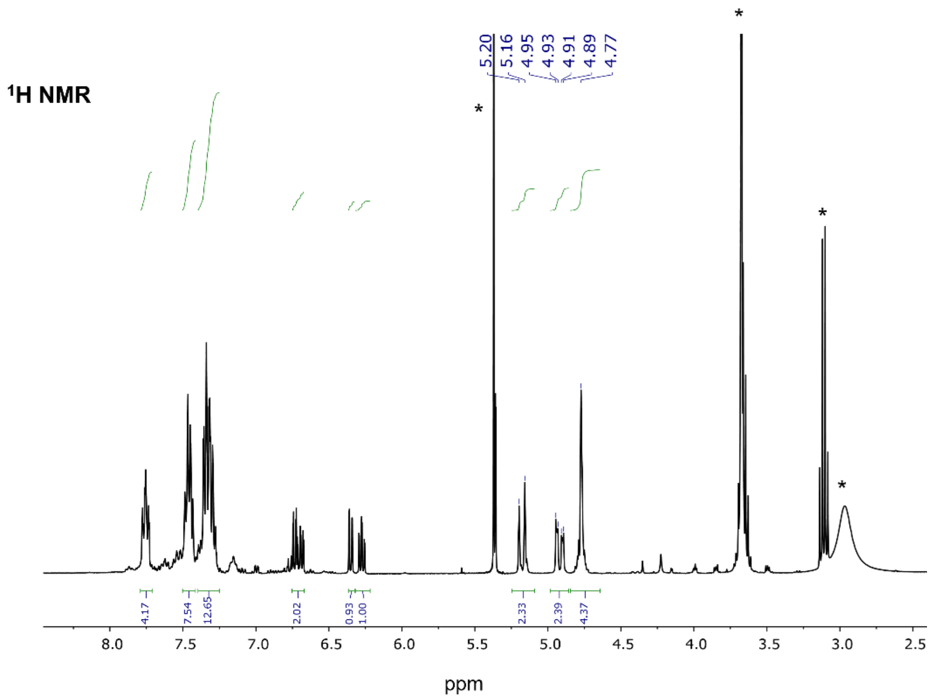

Figure S23: $^{31}P\{^1H\}$ NMR Spectrum (CD_2Cl_2, 298 K, 162 MHz) of [ReO(gly-O,O)(κ^3-$NP_2^{Ph}O^{Ar}$)] (**Re-gly-O,O-NP$_2$O**) (**8**)

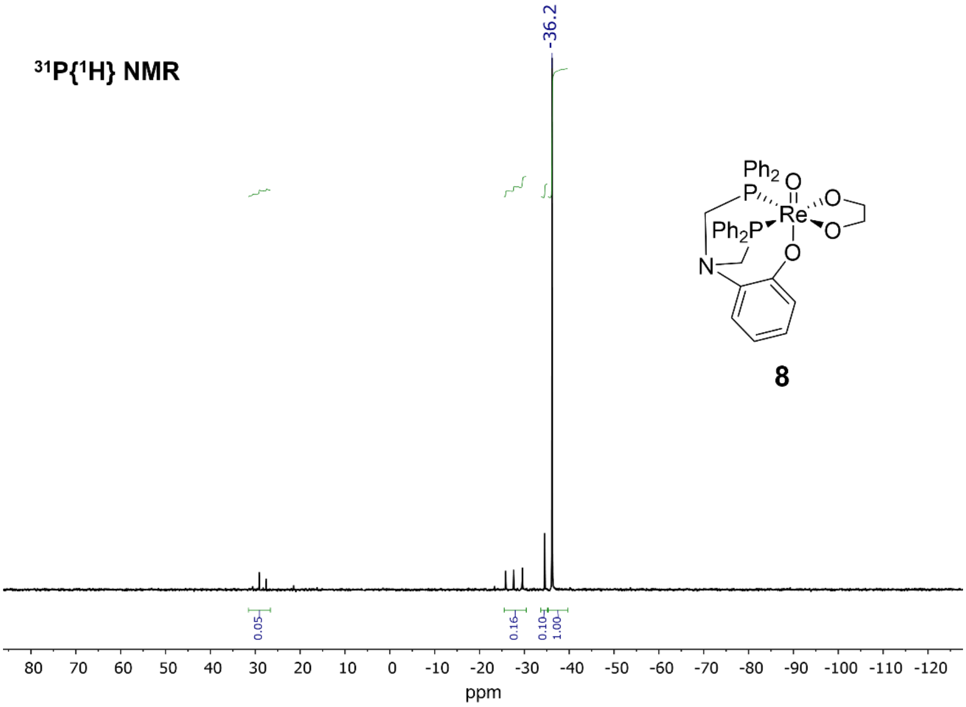

Figure S24: Partial ^{1}H NMR Spectrum ($CDCl_3$, 298 K, 400 MHz) of [ReO(cou-O,O)(κ^3-$NP_2^{Ph}O^{Ar}$)] (**Re-cou-O,O-NP$_2$O**) (**9**). Spectrum below 2.5 ppm with additional solvent impurities has been omitted for clarity.

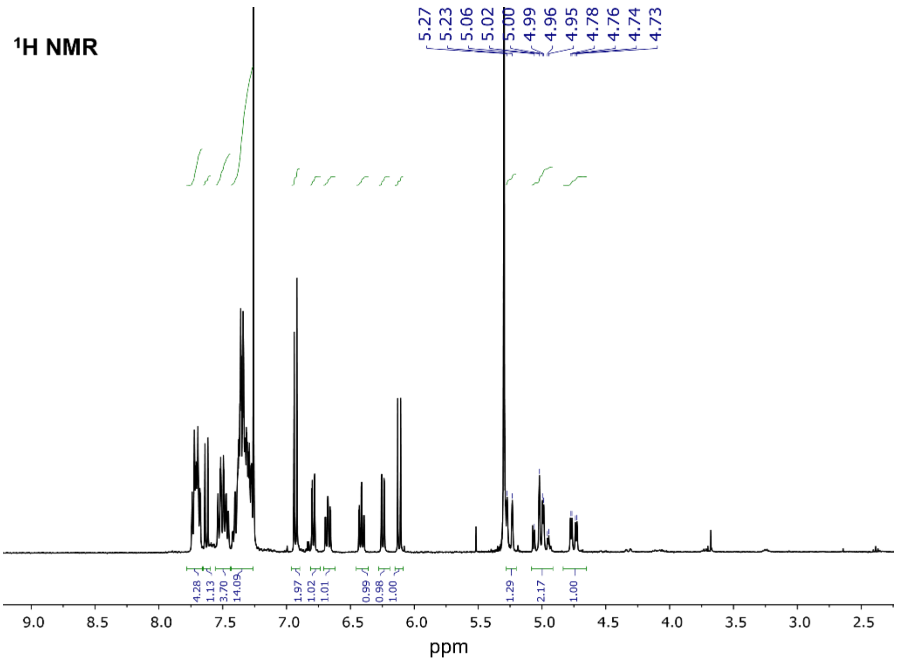

Figure S25: $^{31}P\{^{1}H\}$ NMR Spectrum ($CDCl_3$, 298 K, 400 MHz) of [ReO(cou-O,O)(κ^3-$NP_2^{Ph}O^{Ar}$)] (**Re-cou-O,O-NP$_2$O**) (**9**)

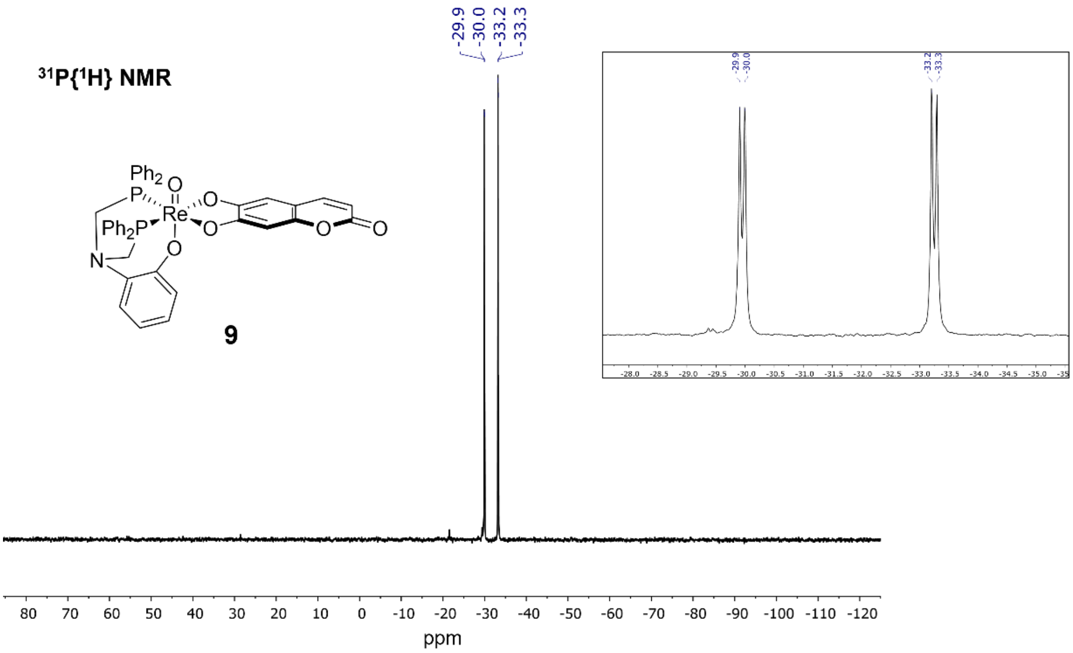

Figure S26: Partial ^{1}H NMR Spectrum of [ReO(dhpma-O,O)(κ^3-$NP_2^{Ph}O^{Ar}$)] (**Re-dhpma-O,O-NP_2O**) (**10**). Spectrum below 2.5 ppm with additional solvent impurities has been omitted for clarity.

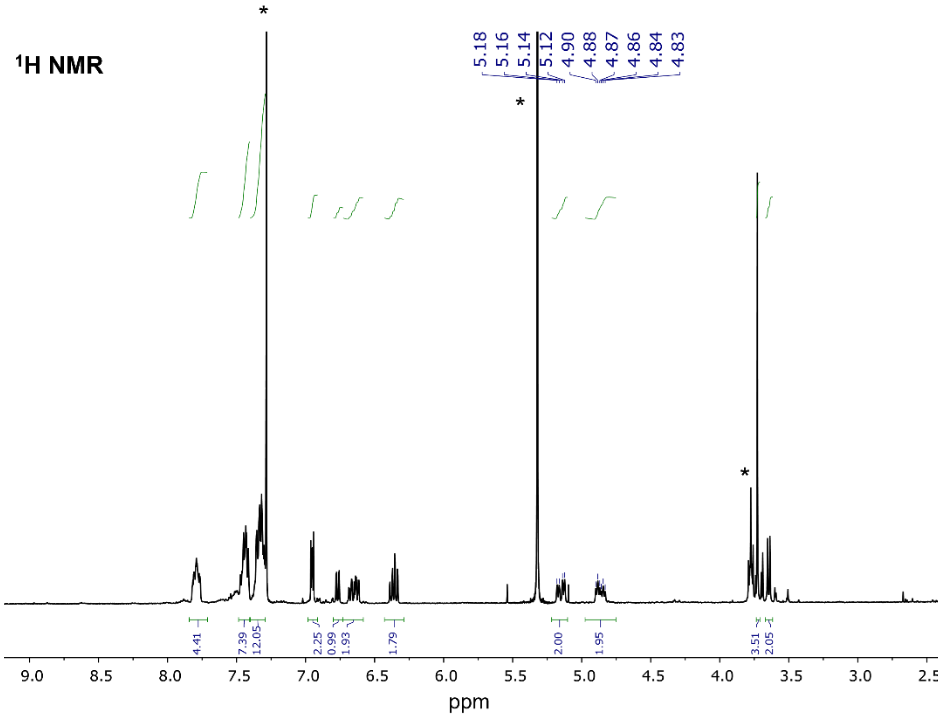

Figure S27: $^{31}P\{^{1}H\}$ NMR Spectrum ($CDCl_3$, 298 K, 400 MHz) of [ReO(dhpma-O,O)(κ^3-$NP_2^{Ph}O^{Ar}$)] (**Re-dhpma-O,O-NP_2O**) (**10**)

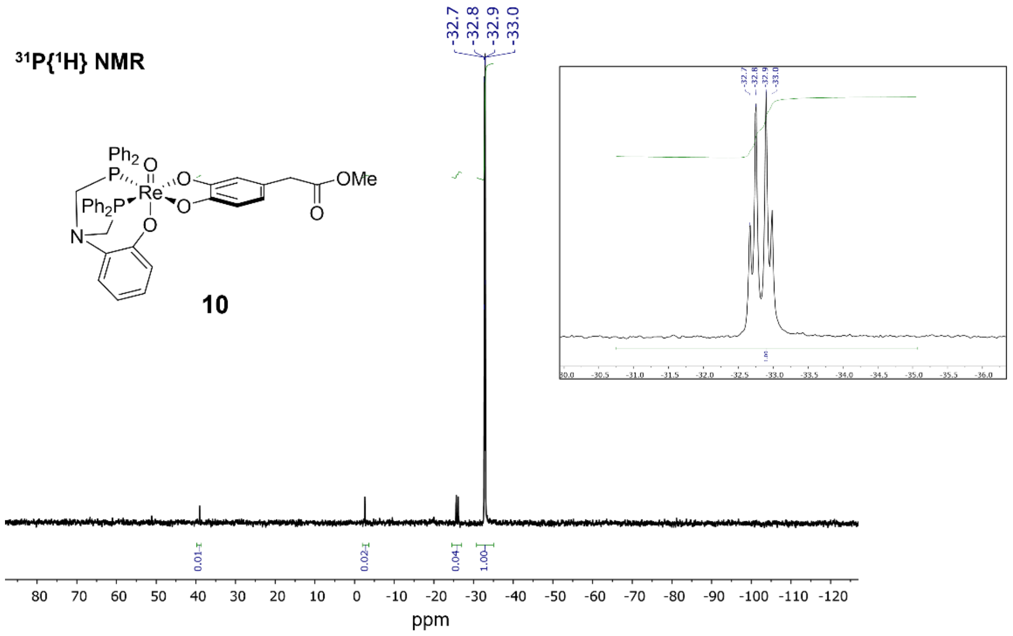

Figure S28: Partial ^{1}H NMR Spectrum ($CDCl_3$, 298 K, 400 MHz) of [ReO(dop-O,O)(κ^3-$NP_2^{Ph}O^{Ar}$)] (**Re-dop-O,O-NP$_2$O**) (**11**). Spectrum below 2.5 ppm with additional solvent impurities has been omitted for clarity.

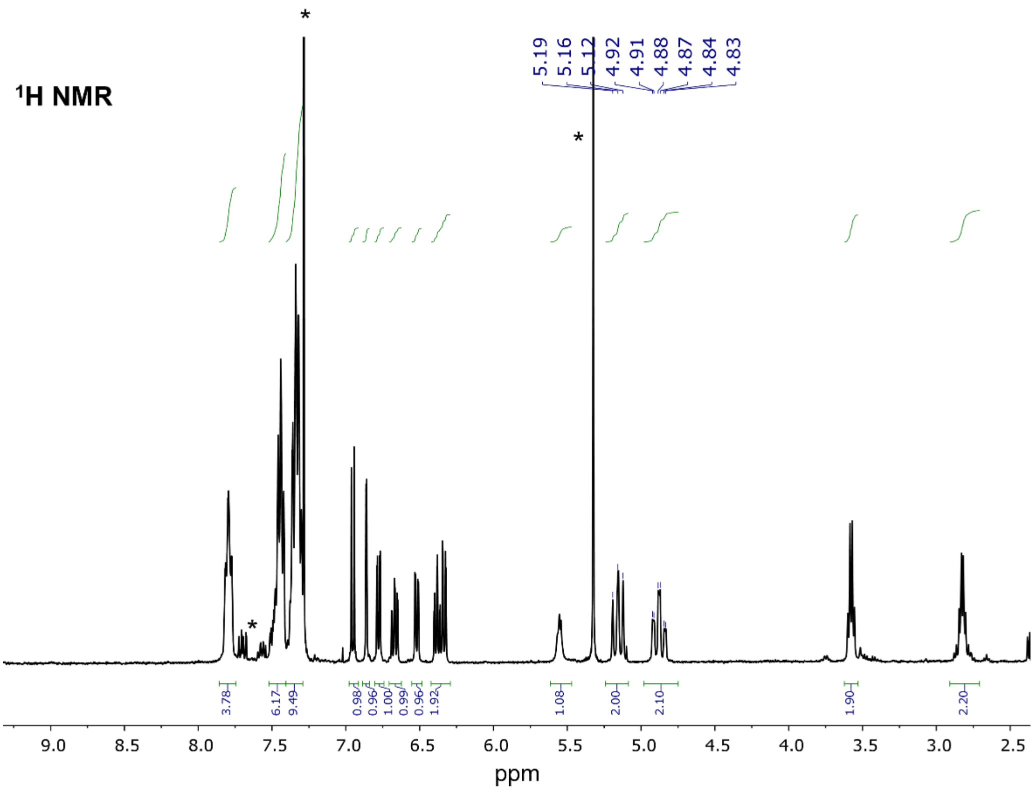

Figure S29: $^{31}P\{^{1}H\}$ NMR Spectrum ($CDCl_3$, 298 K, 400 MHz) of [ReO(dop-O,O)(κ^3-$NP_2^{Ph}O^{Ar}$)] (**Re-dop-O,O-NP$_2$O**) (**11**)

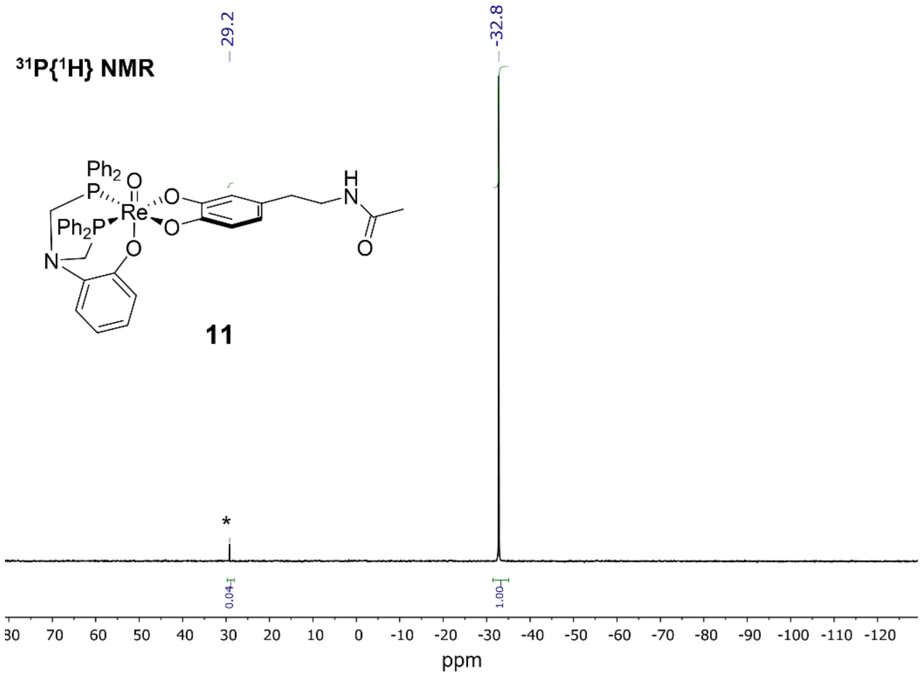

Figure S30: ^{1}H NMR Spectrum ($CDCl_3$, 298 K, 400 MHz) of [ReO(ar-S,O)(κ^3-$NP_2^{Ph}O^{Ar}$)] (**Re-ar-S,O-NP₂O**) (**12**)

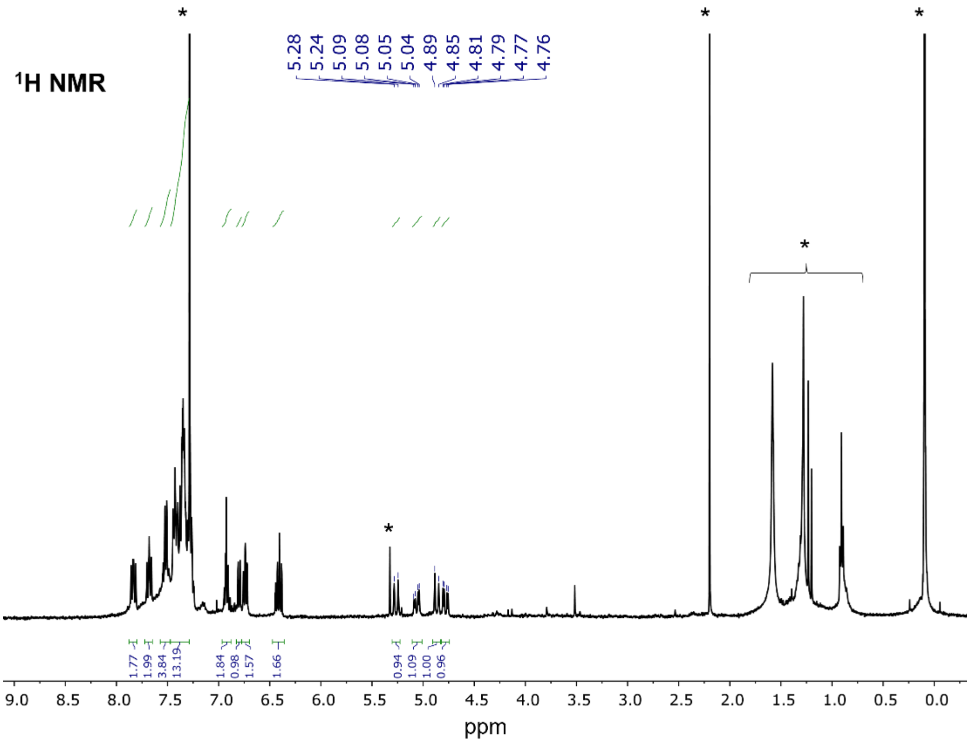

Figure S31: $^{31}P\{^{1}H\}$ NMR Spectrum ($CDCl_3$, 298 K, 400 MHz) of [ReO(ar-S,O)(κ^3-$NP_2^{Ph}O^{Ar}$)] (**Re-ar-S,O-NP₂O**) (**12**)

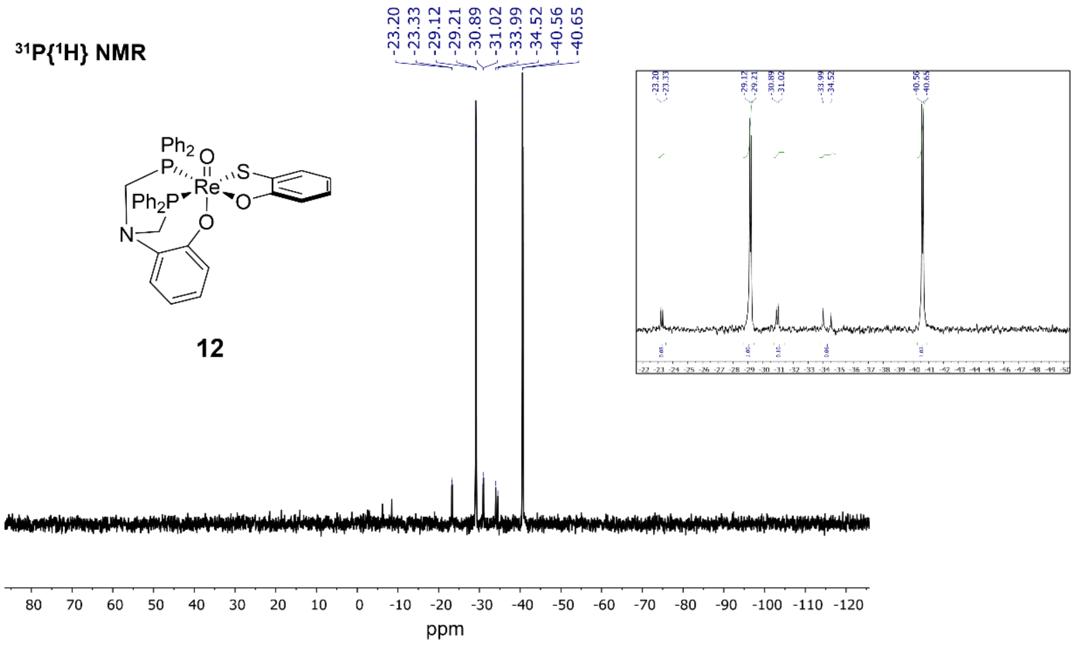

Figure S32: Partial ^{1}H NMR Spectrum (d^{3}-MeCN, 298 K, 400 MHz) of $[TcOCl_3(\kappa^3\text{-}NP_2^{Ph}OH^{Ar})]$ (**Tc-NP$_2$OH**) (**Tc-3**). Spectrum below 4.0 ppm with additional solvent and $[NBu_4]^+$ impurities has been omitted for clarity.

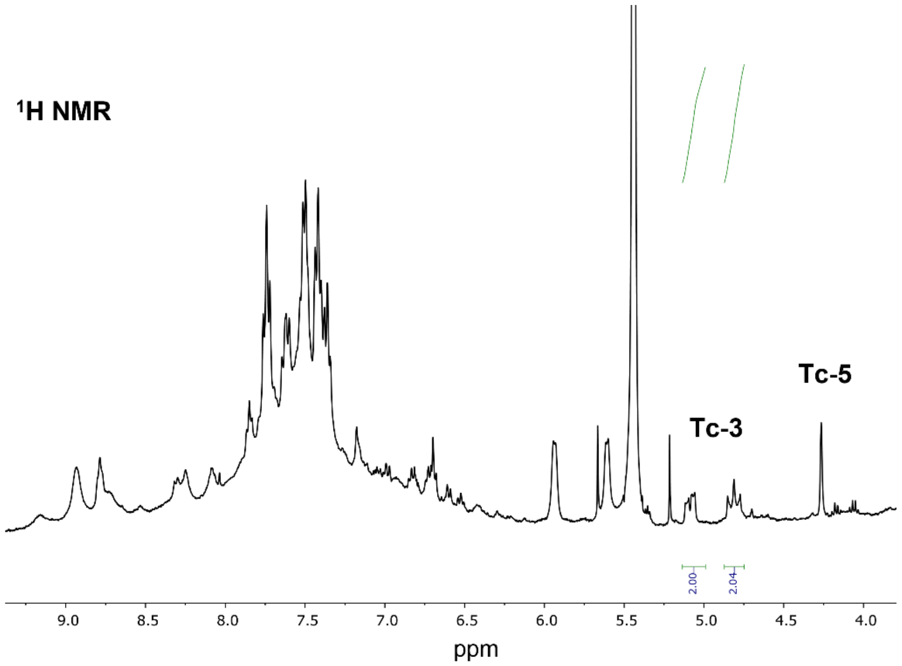

Figure S33: $^{31}P\{^{1}H\}$ NMR Spectrum (d^{3}-MeCN, 298 K, 400 MHz) of $[TcOCl_3(\kappa^3\text{-}NP_2^{Ph}OH^{Ar})]$ (**Tc-NP$_2$OH**) (**Tc-3**)

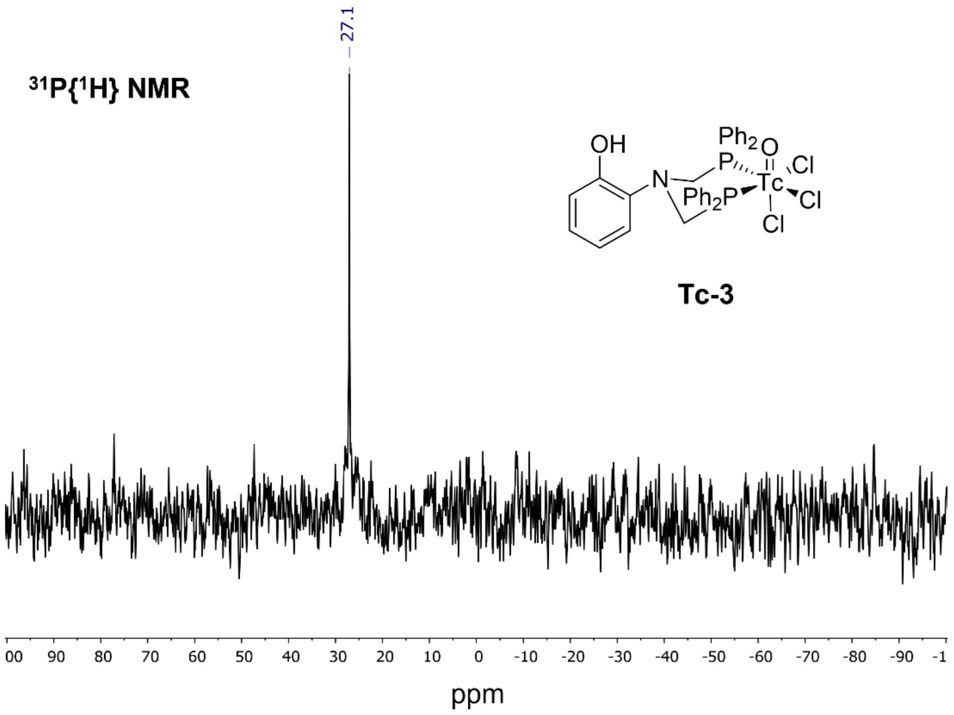

Figure S34: Partial ^{1}H NMR Spectrum (d^3-MeCN, 298 K, 400 MHz) of [TcO(cat-O,O)(κ^3-$NP_2^{Ph}O^{Ar}$)] (**Tc-cat-O,O-NP2O**) (**Tc-6**) & [TcO_2Cl((κ^3-$NP_2^{Ph}OH^{Ar}$) (**TcO_2-NP_2OH**) (**Tc-5**). Spectrum below 4.0 ppm with additional solvent and $[NBu_4]^+$ impurities has been omitted for clarity.

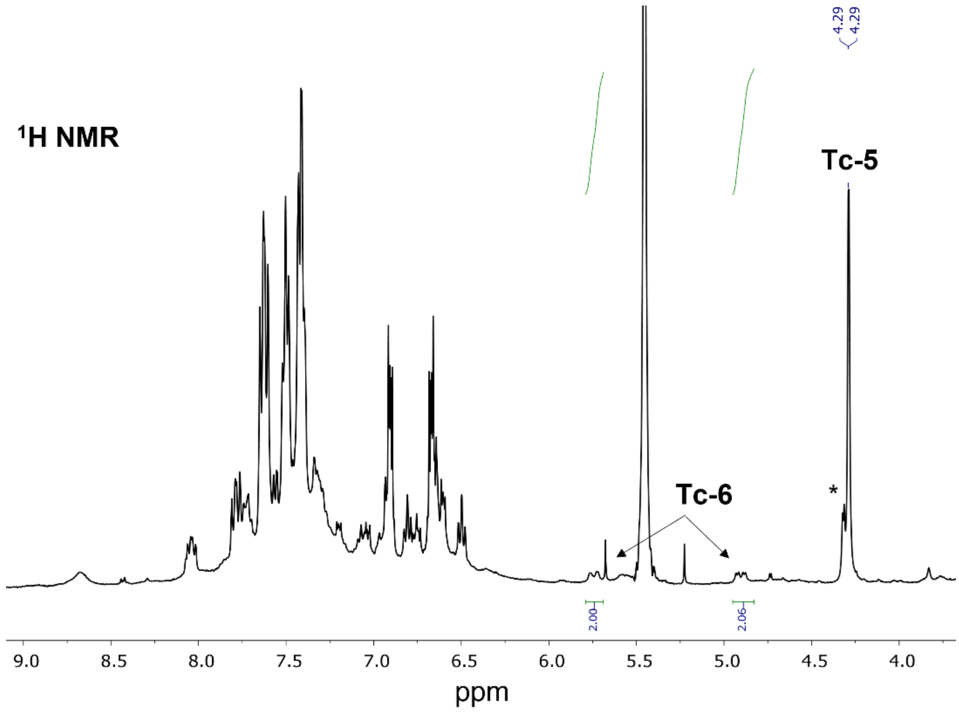

Figure S35: ^{31}P{^{1}H} NMR Spectrum (d^3-MeCN, 298 K, 400 MHz) of [TcO(cat-O,O)(κ^3-$NP_2^{Ph}O^{Ar}$)] (**Tc-cat-O,O-NP2O**) (**Tc-6**) & [TcO_2Cl((κ^3-$NP_2^{Ph}OH^{Ar}$) (**TcO_2-NP_2OH**) (**Tc-5**)

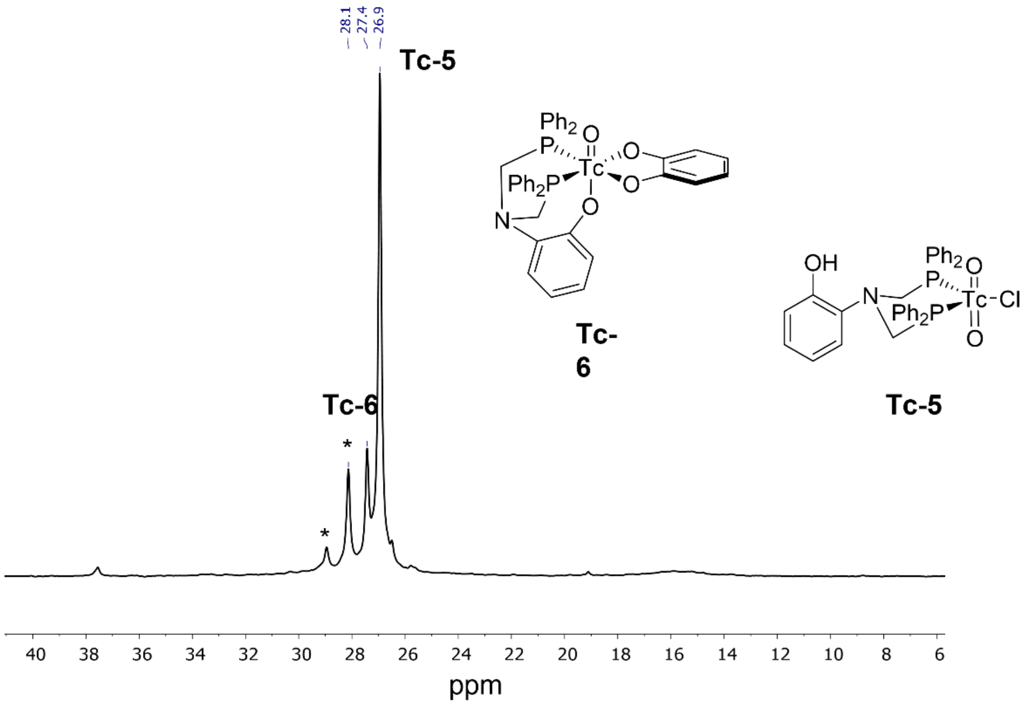

3) ^{99}Tc Complexes TLC Data

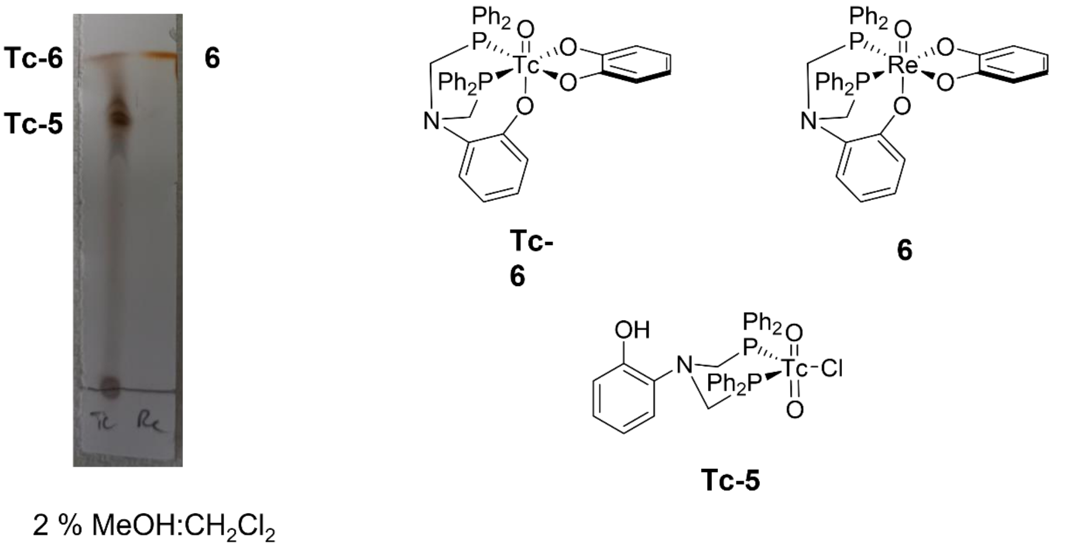

Figure S36: TLC under visible light for reaction products in the attempted synthesis of **Tc-6**.

4) Ligand Synthesis

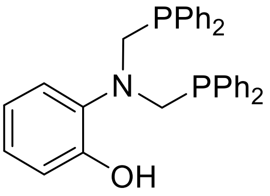

The ligand $NP_2^{Ph}OH$ was synthesised by an adapted synthesis from Durran et a..[4] Paraformaldehyde (0.8 g, 26.85 mmol) was added to a Schlenk flask and diphenylphosphine (5.0 g, 26.85 mmol) added *via* syringe. The reaction mixture was heated to 100 °C for 4 h over which time the mixture turned from cloudy to colourless. 2-Aminophenol (1.45 g, 13.43 mmol) was dissolved in dried and degassed MeOH (50 mL) and transferred into the reaction vessel. Dried and degassed toluene was added (30 mL) and the reaction mixture heated to 60 °C for 18 h. The solvent was removed *in vacuo*, the yellow deposit left was dissolved in minimal CH_2Cl_2, and precipitated from hexane. The precipitate was collected by cannula filtration and dried under high vacuum for 5 h. NMR analysis indicated formation of the desired product in high purity (4.68 g, 69 %).

^{1}H NMR (400 MHz, $CDCl_3$): δ_H /ppm = 7.56 – 7.47 (m, 1H, Ar-*H*), 7.44-7.19 (m, 20H, Ar-*H*), 7.11 – 7.02 (m, 1H, Ar-*H*), 6.83 – 6.76 (m, 2H, Ar-*H*), 5.93 (s, 1H, Ar-O*H*), 3.98 (d, $^{2}J_{HP}$ = 4.4 Hz, 4H, N-CH_2-PPh_2). ^{31}P{^{1}H} NMR (162 MHz, $CDCl_3$): δ_P /ppm = – 25.8 (s, 2P, RPh_2P).

Figure S37: ^{1}H NMR Spectrum ($CDCl_3$, 298 K, 400 MHz) of **$NP_2^{Ph}OH^{Ar}$**

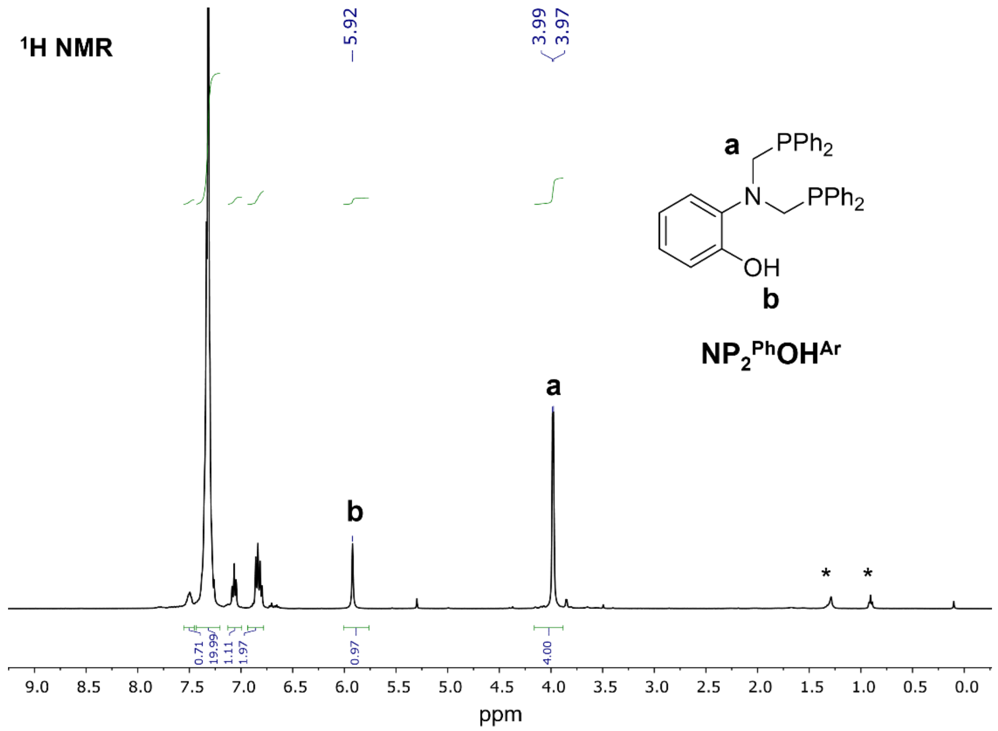

Figure S38: ^{1}P{^{1}H} NMR Spectrum ($CDCl_3$, 298 K, 162 MHz) of **$NP_2^{Ph}OH^{Ar}$**

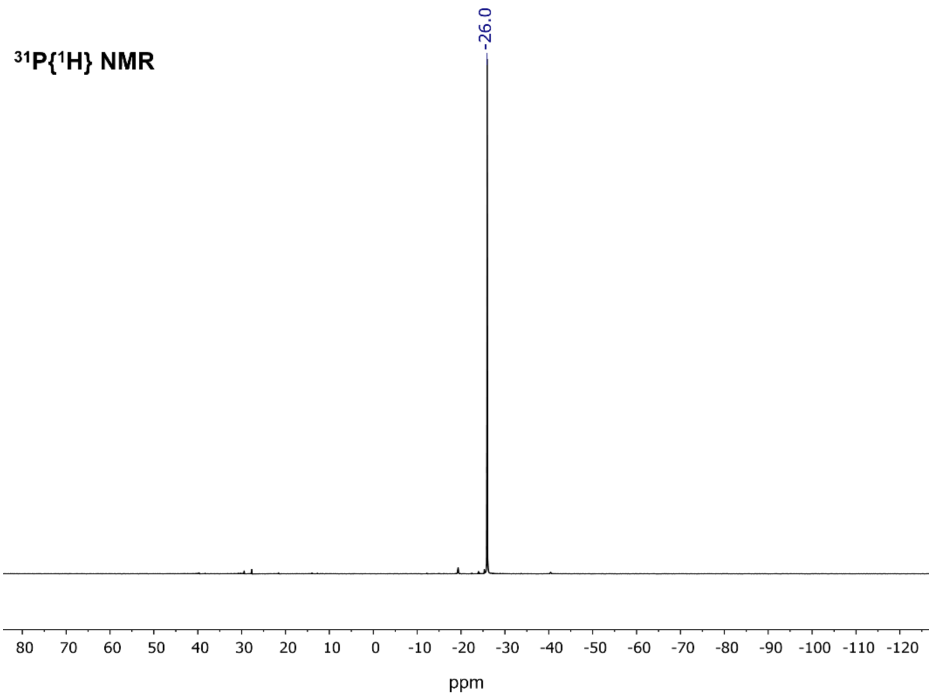

References

[1] SHELXTL v5.1, Bruker AXS, Madison, WI, 1998.

[2] SHELX-2013, Sheldrick, G. M.; *Acta Cryst.*, **2015**, *A71*, 3-8. https://doi.org/10.1107/S2053273314026370.

[3] Spek, A. L.; (2003, 2009) PLATON, A Multipurpose Crystallographic Tool, Utrecht University, Utrecht, The Netherlands. See also Spek, A. L.; *Acta. Cryst.* **2015**, *C71*, 9-18. https://doi.org/10.1107/S2053229614024929.

[4] Durran, S. E.; Elsegood, M. R. J.; Hawkins, N; Smith, M. B.; Talib, S.; *Tet. Lett.* **2003**, *44*, 5255–5257. https://doi.org/10.1016/S0040-4039(03)01274-7.
